# EEG and ECG Wearable Biosensor-Based Affective State Analysis Using Deep Learning over 6G IoT Healthcare Networks

**DOI:** 10.3390/bios16080400

**Published:** 2026-07-23

**Authors:** Hazal Su Bıçakcı Yeşilkaya, Mete Özbaltan, Nihan Özbaltan, Cihat Şeker, Bartu Yeşilkaya, Bengisu Yalçınkaya

**Affiliations:** 1 Department of Electrical and Electronics Engineering, Faculty of Engineering and Architecture, İzmir Bakırçay University, 35665 İzmir, Türkiye; mete.ozbaltan@bakircay.edu.tr (M.Ö.); cihat.seker@bakircay.edu.tr (C.Ş.); 2 Department of Computer Engineering, Faculty of Engineering and Architecture, İzmir Bakırçay University, 35665 İzmir, Türkiye; nihan.ozbaltan@bakircay.edu.tr (N.Ö.); bengisu.yalcinkaya@bakircay.edu.tr (B.Y.); 3 Department of Biomedical Engineering, Faculty of Engineering and Architecture, İzmir Katip Çelebi University, Balatcik Campus, 35620 İzmir, Türkiye; bartu.yesilkaya@ikcu.edu.tr

**Keywords:** wearable biosensors, EEG, ECG, affective state analysis, deep learning, emotion recognition, 6G IoT networks, ray tracing

## Abstract

Physiological signal analysis using wearable biosensors like an electroencephalogram (EEG) and an electrocardiogram (ECG) is widely investigated for affective computing; however, the integration of deep learning-based affective computing within 6G-driven IoT healthcare infrastructures remains limited, with data transmission latency posing a significant challenge. This study proposes a framework for EEG and ECG-based affective state analysis over 6G IoT networks. We utilize an attention-based deep learning model for three-class emotion recognition from EEG signals, and a ResNet50-based convolutional neural network for three-class stress/affective state classification using ECG data. The framework’s communication performance is evaluated through ray tracing simulations in a virtual hospital environment at 7 GHz and 92 GHz bands. Experimental results on SEED and WESAD datasets demonstrate that the EEG model achieved 90.93% classification accuracy, while the ECG model yielded 82.26% validation and 65.64% test accuracy. Wireless analysis showed RMS delay spread values of 6.53 ns (7 GHz) and 3.32 ns (92 GHz), with correlation bandwidths of 30.63 MHz and 60.24 MHz, respectively. These findings demonstrate the feasibility of integrating wearable biosensor-based affective state analysis with 6G-oriented IoT healthcare communication frameworks, providing a robust foundation for future personalized health and human state monitoring applications.

## 1. Introduction

Wearable biosensor-based healthcare monitoring systems and physiological signal analysis have been extensively investigated for continuous and real-time monitoring, affective computing, and intelligent healthcare applications [1,2]. Physiological signals acquired from biosensors such as an electroencephalogram (EEG) and an electrocardiogram (ECG) provide valuable information regarding neurological and cardiovascular activity, enabling the development of data-driven healthcare monitoring systems [3]. In addition, recent advances in deep learning (DL) and IoT-enabled healthcare infrastructures have further increased interest in wearable biosensor-based affective state analysis and remote healthcare applications [1,2,4]. However, the reliable and low-latency transmission of physiological data remains an important challenge for real-time healthcare monitoring systems, particularly in large-scale IoT environments. In this context, next-generation 6G IoT networks are expected to support high-speed, reliable, and scalable physiological data transmission for future healthcare applications [5].

The rapid development of IoT technologies has enabled the continuous monitoring and transmission of physiological signals, such as EEGs and ECGs, which have significantly contributed to remote healthcare and wearable biosensor-based monitoring systems. Recent studies have investigated the integration of real-time cognitive and affective state-aware frameworks into IoT-enabled healthcare applications [1,2]. These studies also emphasize that communication networks with scalability and low-latency capabilities are critical for reliable physiological data transmission in such applications. In this context, 6G networks are expected to further enhance real-time IoT-based healthcare monitoring systems by providing ultra-low latency, massive device connectivity, high reliability, and extremely high data rates [5].

Based on our literature review, studies integrating biosensor-based affective state analysis with real-time IoT-enabled healthcare communication infrastructures remain relatively limited, particularly for EEG/ECG data transmission and processing over next-generation 6G networks. Existing studies generally focus either on physiological signal analysis or healthcare communication frameworks separately and are often evaluated under limited application scenarios. Therefore, the main motivation of this study is to investigate the integration of EEG/ECG-based affective state analysis with 6G-oriented IoT healthcare communication systems within a unified framework.

In this study, a framework for EEG and ECG wearable biosensor-based affective state analysis using DL over 6G IoT networks is proposed. For accurate analysis of affective states, EEG data are processed using an attention-based DL model, while ECG data are analyzed using a ResNet50-based convolutional neural network (CNN). To support reliable and low-latency physiological signal transmission, ray tracing-based wireless communication modeling is performed for 6G IoT network environments. In this way, the proposed framework aims to support real-time healthcare monitoring and physiological signal-based assessment applications.

The main novelty of this study is the presentation of an integrated framework for EEG and ECG wearable biosensor-based affective state analysis over 6G IoT healthcare networks, which differs from existing studies that generally focus on physiological signal analysis or healthcare communication frameworks separately. Another important aspect of this study is the combined use of EEG and ECG signals, as these biosignals are widely utilized and reliable indicators for monitoring physiological and affective states. An attention mechanism was leveraged to capture the temporal dynamics of EEG signals, while ResNet50 was utilized to extract distinctive morphological features from ECG signals. In addition, a hybrid DL framework compatible with the characteristics of both biosignals was proposed to improve the performance of existing approaches in the literature. Furthermore, this study was designed within a framework that leverages the low-latency capabilities of 6G networks and incorporates ray tracing-based wireless communication modeling for realistic healthcare environments. Therefore, the proposed framework demonstrates the feasibility of real-time biosensor-based healthcare monitoring applications.

Contributions: The main contributions of the proposed framework are listed as follows:

- Framework: We propose a framework for EEG and ECG wearable biosensor-based affective state analysis using DL over 6G IoT healthcare networks.
- Hybrid wearable biosensor-based DL model for affective state analysis: EEG signals are processed using an attention-based model, while ECG signals are analyzed with a ResNet50-based CNN for effective feature extraction and improved classification performance.
- 6G IoT-based ray tracing model: Wireless transmission of EEG and ECG data is analyzed for 6G IoT networks using ray tracing simulations in a virtual hospital room scenario, evaluating the path loss, RMS delay spread, and correlation bandwidth for FR3 and mmWave frequency bands.

The outline of this paper is as follows: The next section provides a comprehensive literature review. Section 3 presents the technical background on EEG and ECG signals, deep learning, and 5G/6G IoT networks. Section 4 details the proposed EEG and ECG wearable biosensor-based affective state analysis framework over 6G IoT healthcare networks. Section 5 presents the experimental evaluations. Finally, this paper concludes with Section 6.

## 2. Related Work

Physiological signals collected from wearable biosensors, including ECGs and EEGs, are frequently used in e-health applications [4]. The ability of e-health applications to process incoming data and transport this data from patients to users swiftly and accurately has vital importance [6]. The signals in the data are also frequently utilized for emotion recognition purposes [3,7,8], which is a crucial component in the development of artificial intelligence (AI)-supported applications. In addition, the advent of next-generation 6G IoT networks promises to provide the rapid, reliable, and high-capacity transmission of biosensor-based physiological signals, thus offering a significant enhancement to real-time healthcare applications [9].

Accordingly, this section first proposes an overview of physiological signal-based emotion recognition studies along with the benchmark datasets in order to establish the broader context of affective state analysis using wearable biosensors. It then specifically focuses on EEG- and ECG-based emotion recognition applications, respectively. Finally, studies addressing 6G IoT networks for remote healthcare systems are reviewed to discuss the communication requirements associated with the reliable and low-latency transmission of biosensor-derived physiological data in real-time healthcare monitoring environments.

Physiological signal-based emotion recognition has evolved from conventional feature engineering approaches to DL and multimodal biosensing frameworks [10]. Reviews in this field have shown that EEG and peripheral physiological signals, particularly ECG or heart rate variability (HRV), electrodermal activity, respiration, and skin temperature, provide complementary information regarding affective responses [11]. These signals also bring some practical challenges such as subject dependence, sensor placement, distortions caused by motion, and the requirements of real-time monitoring [12,13,14,15]. Hui and Sherratt [16] developed a wearable emotion recognition framework combining photoplethysmography (PPG), electrodermal activity (EDA), skin temperature, and electromyography (EMG) measurements synchronized with audiovisual stimuli. Their results highlighted the importance of selecting the physiological response interval corresponding to the elicited affective episode. Benchmark datasets have played an important role in enabling systematic evaluation of these approaches. In addition to the DEAP, SEED, and WESAD datasets frequently used in the literature, MAHNOB-HCI, DREAMER, ASCERTAIN, and AMIGOS provide multimodal recordings involving EEG, ECG, or other wearable physiological signals under valence–arousal or discrete affective state settings [17,18,19,20].

For EEG-based emotion recognition, early studies mainly relied on handcrafted spectral, asymmetry, and statistical features combined with conventional machine learning classifiers. Jenke et al. [21] investigated feature extraction and electrode position selection strategies for EEG emotion recognition, while Zheng et al. [22,23] examined stable EEG patterns over time and multichannel feature relationships using group-sparse canonical correlation analysis. Since EEG responses may vary considerably across subjects and sessions, domain adaptation approaches have also been proposed to reduce cross-domain performance degradation [24]. With the increasing availability of benchmark datasets, DL methods have become more prominent [25]. Hierarchical CNNs, hybrid deep neural networks, convolutional recurrent architectures, and four-dimensional convolutional recurrent models have been used to learn spatial and temporal representations directly from EEG signals or their structured transformations [26,27,28,29,30,31]. More recent approaches involve electrode topology, temporal asymmetry, and attention mechanisms. Dynamical graph CNNs, regularized graph neural networks, temporal and spatial architectures, and attention-based recurrent models have reported improved emotion recognition performance [32,33,34,35,36,37]. Qin et al. [38] reviewed EEG acquisition and feedback systems from electrode selection and signal pre-processing to classification and real-time feedback, emphasizing that reliable EEG-based applications depend on the complete acquisition and processing chain. More recently, Yan et al. [39] proposed a multi-view EEG–fNIRS architecture that modeled frequency domain properties of an EEG together with temporal and spatial information and fused the two methods through a cross-attention mechanism. On a four-class dataset involving 50 participants, the authors reported an average accuracy of 89.44% for the EEG alone and 96.09% for the EEG–fNIRS fusion.

In parallel with EEG-based studies, ECGs and other physiological signals have been increasingly employed for affective state and stress recognition. Multimodal frameworks such as EmotionMeter have demonstrated that complementary physiological and behavioral measurements can improve affect analysis. Datasets such as DREAMER and AMIGOS enable the analysis of ECG-based affective responses in combination with EEGs and other signals [18,20,40]. ECG-oriented studies have investigated affective information through HRV features, temporal patterns, and transformed signal representations. Nardelli et al. [41] examined emotions induced by affective sounds using HRV-based measures, Domínguez-Jiménez et al. [42] developed a machine learning (ML) model based on physiological signals for emotion recognition, and Sepúlveda et al. [43] employed wavelet scattering and machine learning for emotion recognition from ECG signals. Wearable sensing studies have also addressed continuous stress and affect monitoring under more practical settings, demonstrating the relevance of physiological signal-based models for real-life and healthcare-oriented applications [44,45]. Rinella et al. [46] examined HRV features derived from ECG signals together with pulse rate variability and pulse shape features from PPG for affect recognition. Using KNN and SVM classifiers, they reported accuracies generally ranging from 50% to 72%, depending on the affective variable, feature group, and classifier. Their results showed that features obtained from PPG could provide performance comparable to ECG features and that the pulse structure contained additional information associated with affective responses.

Although these studies provide substantial advances in emotion recognition accuracy, wearable sensing, and multimodal physiological analysis, their primary emphasis is generally placed on affective state inference and dataset-level evaluation. In healthcare monitoring scenarios, however, the recognition model must also be supported by a communication infrastructure capable of transmitting physiological data reliably and with sufficiently low latency. In the studies reviewed above, the wireless propagation behaviour of EEG- and ECG-related biosensor data is generally not evaluated together with the affective state recognition task in an indoor clinical environment. Therefore, the present study complements existing emotion recognition frameworks by considering modality-specific DL models for physiological signal-based affective state analysis together with a ray tracing-based 6G-oriented IoT communication scenario in a virtual hospital environment.

### 2.1. EEG-Based Emotion Recognition

An EEG is a method for non-invasively measuring electrical activity in the brain via electrodes placed on the scalp. This method provides insights into continuous and changing neuronal interactions. The emergence of wireless technologies, such as Wi-Fi and Bluetooth, have made it possible to collect EEG data using portable devices, thereby enabling extensive research in the field of brain–computer interface (BCI) systems and cognitive science applications. To advance BCI systems, EEG signals are utilized in emotion estimation studies, where they offer critical diagnostic and research value, as they can detect changes in brain activity associated with emotional states [47,48,49,50,51]. Emotional modeling in these studies is executed through two predominant methodologies. The first model is the discrete emotion model, which divides affective states into six categories: fear, sadness, hate, surprise, nervousness, and happiness. The second model is the dimensional emotion model [52], which is commonly represented by the valence/arousal (VA) space. Within this framework, valence is defined as the measure of positive or negative emotional response, whereas arousal is defined as the intensity of that emotional response, ranging from low-activation states of calmness to high-activation states of excitement.

The recognition of emotion utilizing EEG signal content involves considerable challenges. These challenges occur due to the brain’s complexity in cognitive functions and the irregular nature of EEG data. A series of studies have investigated a variety of AI methodologies for emotion classification based on EEG signals stimulated by visual or audio stimuli. Liu et al. [53] utilized the IAPS dataset, obtaining accuracies of 75.53% and 79.19% in valence and arousal classification, respectively, with the usage of power spectral features and KNN. Mehmood and Lee [54] used portable EEG devices with independent component analysis, Hjorth parameters, and metaheuristic optimization to achieve results, with a maximum of 57.42% accuracy. Mohammadpour et al. [55] conducted a comparative analysis of multiple classifiers, achieving an accuracy of approximately 55% through the implementation of the discrete wavelet transform and PCA. Krisnandhika et al. [56] developed a neural network-based system based on a radial basis function, observing improved accuracy with larger sample sizes. Candra et al. [57] applied wavelet decomposition and SVM algorithms. Their results obtained better performance in valence classification and identified key EEG channels (e.g., F3, F4, Fz) associated with emotional responses. Yeşilkaya et al. [8] conducted a gender-dependent study using 20 female and 20 male subjects’ data with KNN, subspace KNN ensemble, and SVM classifiers. They indicated that valence and arousal parameters can be classified better in women than men in all cases. In women, the best results were obtained with the KNN classifier, with an arousal classification accuracy of 69.1%, while the valence accuracy was 69.4% with the ensemble classifier. In men, the highest arousal accuracy was obtained with the KNN classifier at 65.1%, while the valence accuracy was 66.1% with the SVM classifier.

### 2.2. ECG-Based Emotion Recognition

In comparison with the results presented by Schmidt et al. [58] in the first WESAD study, where the average accuracy was found to be around 93% for the two-class stress vs. non-stress classification and 80% for the three-class classification using traditional ML methods, namely, Random Forest, AdaBoost, and Linear Discriminant Analysis, the proposed deep learning model is evaluated on a comparable three-class subject-wise split. Bıçakcı et al. [3] used the same dataset for three-class classification problems with Decision Trees, SVM, KNN, Bagged Tree Ensemble, subspace KNN ensemble and neural network models. They achieved overall accuracy rates between 53.71% and 93.67% for different feature sets and classifiers. The proposed model distinguishes the three affective states (baseline, stress, and meditation) under a subject-independent evaluation, which avoids subject-specific leakage between training and test sets.

Further research, including DL techniques, has shown incremental results on the WESAD dataset, though none have been able to replicate the results obtained with this current model. For instance, Gjoreski et al. [59] used a CNN to perform a binary classification task, obtaining an accuracy rate of around 95%, while Seo et al. [60] used a combination of a CNN and LSTM, obtaining around 96% accuracy in a binary classification task. Other research, including the use of LSTM, such as Can et al. [61], obtained an accuracy rate of around 92% in a three-class task. In our experiments, the proposed ResNet50-based branch achieved 82.26% validation accuracy and 65.64% test accuracy on the three-class WESAD setting under a subject-wise split.

It should be noted that the reported ECG classification accuracy in this study refers to the three-class stress/affective state recognition task on the WESAD dataset under the employed experimental configuration (82.26% validation and 65.64% test accuracy in our subject-wise evaluation section), rather than a clinical diagnostic accuracy of ECG interpretation.

### 2.3. 6G IoT Networks for Remote Healthcare Systems

Recent advancements in IoT technology have led to the formation of modern remote healthcare systems. These systems generally use wearable biosensors, wireless communication networks, and cloud or edge computing platforms to collect, transmit, and analyze patients’ physiological signals, such as EEGs and ECGs, in real time [1,62,63].

In real-time remote healthcare services, efficient transmission of physiological signals requires a network infrastructure that ensures a high data rate, high reliability, and low latency [64,65]. Yaqoob et al. [2] highlighted the potential of AIoT-based emotion recognition systems. They discuss the integration of IoT infrastructures and deep learning models to support scalable emotion-aware healthcare applications using multimodal signals such as speech, facial gestures, EEGs, and ECGs. They also emphasize that the development of real-time emotion-aware healthcare systems highly depends on the employment of scalable and low-latency communication networks.

Security and privacy are also addressed as critical concerns in IoT-based healthcare systems because physiological signals contain highly sensitive personal data. In the Islam et al. [66] study, for secure transmission of biomedical data, including EEGs and ECGs, an adaptive federated learning framework is designed for privacy-preserving collaboration among distributed IoMT devices. Their system integrates edge–fog–cloud architectures and introduces gradient compression mechanisms, reducing communication overhead by 45.3%.

Recently, with the increasing demand for real-time medical services, research has shifted to next-generation communication networks to provide efficient wireless communication in IoT-enabled healthcare applications. In the Alshammari et al. [67] study, the role of 5G networks is investigated in supporting healthcare services during the COVID-19 pandemic. The findings indicate that the AI-based 5G IoT employment can increase the effectiveness of healthcare services such as infection detection, remote diagnosis, and real-time health monitoring. Mahmmod et al. [68] presented an extensive review of IoT-based patient monitoring systems (PMS). In PMS, biomedical data, including ECGs and EEGs, are first transmitted to gateway devices and then forwarded to remote servers through wireless communication, including Wi-Fi and 5G, to achieve real-time monitoring and medical decision support. They highlighted that employing IoT-based PMS and 5G communication networks together can significantly improve data transmission efficiency for healthcare monitoring systems, yet the rapid increase in the amount of physiological data and the number of medical devices in the network causes new challenges for existing communication technologies.

Nowadays, 6G networks are evaluated as a promising solution for developing enhanced IoT-based healthcare systems. The literature predicts that 6G IoT can provide ultra-low-latency communication, massive device connectivity, and extremely high data rates [5]. Chen et al. [69] state that 6G networks can provide a data speed of up to 1 Tbps with a latency time below 0.1 ms. For smart health services, the effectiveness of AI-based 6G applications is also examined in the literature. Sakthi et al. [70] propose ensemble DL methods to detect malicious users accessing electronic health records in 6G networks. A smart healthcare cyber-physical system combining IoT devices, blockchain security, and ML techniques in a cloud-6G network is introduced. They reported 93% privacy protection and, approximately, a 55 ms communication delay. Moreover, Dall’Ora et al. [9] introduced a healthcare system integrating wearable sensors, AI-based behavioral analysis, and emerging 6G communication capabilities to support continuous monitoring and assistance for Alzheimer’s patients.

To sum up, current mobile networks, particularly 5G architectures, represent potential for large-scale medical IoT deployments requiring reliable and prompt communication to a certain extent. High-precision applications, such as real-time remote healthcare systems, require massive machine-type communication (mMTC) and ultra-reliable low-latency communication (URLLC) capabilities offered by emerging 6G networks. Ultra-low latency, a high data rate, and high reliability of 6G IoT networks have been extensively investigated in prior studies at the system level. However, existing studies primarily focus on system architectures along with artificial intelligence models and data security mechanisms in IoT-based healthcare systems. Comparatively little attention has been given to the wireless propagation characteristics of physiological signal transmission, especially within emerging 6G communication frameworks. In this context, our work investigates the transmission characteristics of EEG and ECG signals in a simulated hospital environment using a ray tracing-based 6G communication model. By analyzing signal propagation and path loss characteristics in an indoor clinical scenario, we aim to provide deeper insights into the communication performance of next-generation IoT-enabled remote healthcare systems.

## 3. Technical Background of the Proposed Framework

In this section, the technical foundations for the biosensor-based affective state analysis framework are presented. In this context, an EEG provides information related to emotion-associated neural activity, whereas an ECG and related chest-worn physiological recordings provide autonomic changes associated with stress and affective responses. These biosensor signals can be analyzed using deep learning models for emotion recognition.

Accordingly, this section first presents the physiological basis and analytical characteristics of the EEG and ECG biosensor signals employed in this study. It then describes the deep learning components used to extract affective state-related information from these data. Finally, the communication background is introduced as a supporting component of the biosensor framework, since reliable and low-latency transmission is required for the practical use of continuously acquired physiological data in IoT-enabled healthcare monitoring environments.

### 3.1. EEG

EEG signals are often categorized into five principal frequency bands during signal analysis. The bands of interest in this study include: the delta band (δ), which ranges from 0.5 to 4 Hz; the theta band (θ), which ranges from 4 to 8 Hz; the alpha band (α), which ranges from 8 to 13 Hz; the beta band (β), which ranges from 13 to 30 Hz; and the gamma band (γ), which ranges from 30 to 100 Hz [71]. Each band is connected to specific neurophysiological and cognitive states. δ bands are dominant during deep non-REM sleep and indicate reduced mental activity. θ bands are commonly observed during light sleep and meditative states, and they are related to memory and emotional processing. α bands appear during relaxed wakefulness. β bands are associated with active thinking and sustained attention, while γ bands are linked to higher-level functions, such as perception, learning, and memory consolidation [71].

These rhythms present as spectral patterns that vary over time. Therefore, information related to emotional states is often examined through the evaluation of changes in power within specific frequency bands. Conventional analyses depend on engineered features, such as band power measures, asymmetry indices, and various statistical descriptors. These features are then processed with ML classifiers. While these methodologies can prove effective under controlled conditions and their reliability can be compromised by variations among subjects, the addition of measurement noise, and the dynamic nature of physiological signals.

**Figure 1:**
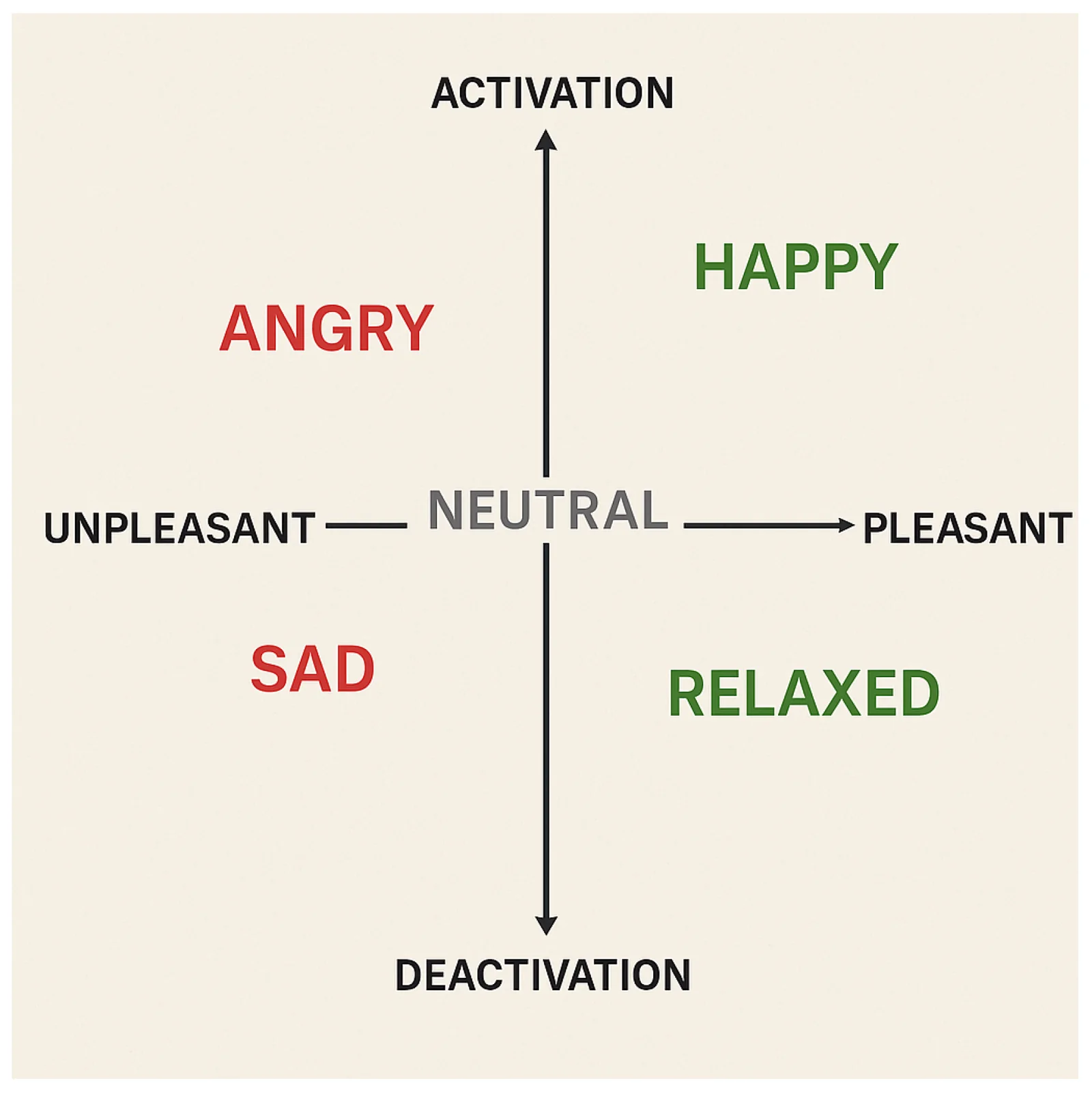
Two-dimensional emotional model.

Russell [52] categorized 28 emotions in a two-dimensional scale with eight categories, such as arousal, pleasure, misery, etc. In our experiment, positive, negative, and neutral emotion categories are investigated. The two-dimensional valence–arousal emotional model is given in  Figure 1. The side of happiness and relaxation represents positive emotions, while the side of anger and sadness represents negative emotions. Neutral emotions are shown in the central position of the figure.

In the literature, the model achieves higher classification performance for the positive and negative emotional states, whereas the neutral emotional state is relatively more difficult to distinguish [72]. This behavior can be attributed to several factors related to the characteristics of emotional responses and EEG signal patterns. Positive and negative emotions generally produce stronger neurophysiological responses and higher emotional arousal compared to neutral states. Consequently, the EEG patterns associated with these emotions tend to exhibit more distinctive characteristics, making them easier for ML models to identify. Unlike positive and negative emotional states, neutral emotional states are associated with reduced emotional intensity and diminished cortical activation, leading to less discriminative EEG features. The neural patterns of neutral states often overlap with those of positive and negative emotions in the feature space, which complicates classification. Emotional responses to neutral stimuli vary across individuals. This variability can hinder the ability of DL models to learn stable feature representations for the neutral class. As a result the lower classification performance observed for the neutral emotional state is likely due to its ambiguity and the overlapping nature of EEG patterns across emotional categories.

### 3.2. ECG

**Figure 2:**
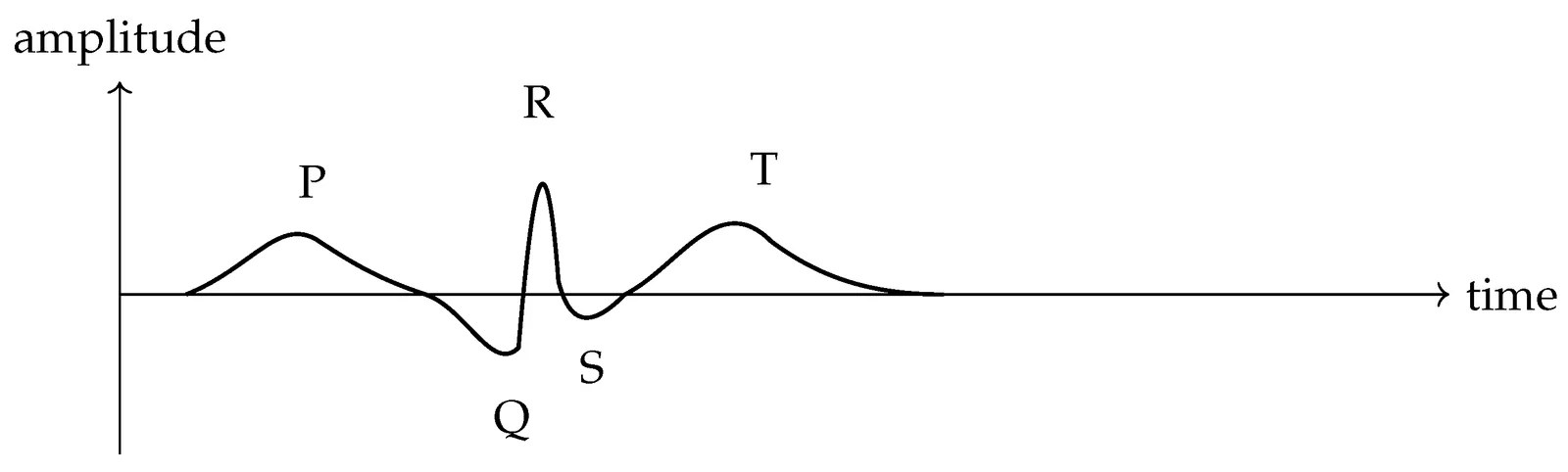
Illustration of a typical ECG beat morphology (P–QRS–T).

Electrocardiography is a method for monitoring the electrical activity of the heart and is an indirect method for assessing autonomic nervous system control. A standard electrocardiogram beat is composed of a P wave, a QRS complex, and a T wave, as illustrated in  Figure 2. Changes in the characteristics of these components reflect changes in autonomic nervous system control, which in turn affect heart rate and beat-to-beat variability. Thus, electrocardiography is an extensively used method for stress and affect analysis.

In practice, affect recognition using electrocardiography can be performed using segments of the electrocardiogram signal or through the use of heart rate variability features derived using the detected R-peaks. We denote the time index for the *i*th detected R-peak as $t_{i}$, and the sequence of RR-intervals is given as $RR_{i}=t_{i}-t_{i-1}$. The instantaneous heart rate is estimated using the RR-intervals as in Equation (1), and a standard time-domain heart rate variability feature is given as RMSSD in Equation (2).

Let $t_{i}$ denote the time index of the detected *i*th R-peak. The corresponding RR-interval sequence is defined as $RR_{i}=t_{i}-t_{i-1}$. From RR-intervals, the instantaneous heart rate (HR) can be approximated as

(1) $$\mathrm{HR}=\frac{60}{\bar{RR}},$$

where $\bar{RR}$ denotes the mean RR-interval (in seconds) over a window. A commonly used time-domain HRV metric is the root mean square of successive differences (RMSSD):

(2) $$\mathrm{RMSSD}=\sqrt{\frac{1}{N-1}\sum_{i=2}^{N}{\left(RR_{i}-RR_{i-1}\right)}^{2}}.$$

### 3.3. Deep Learning

**Figure 3:**
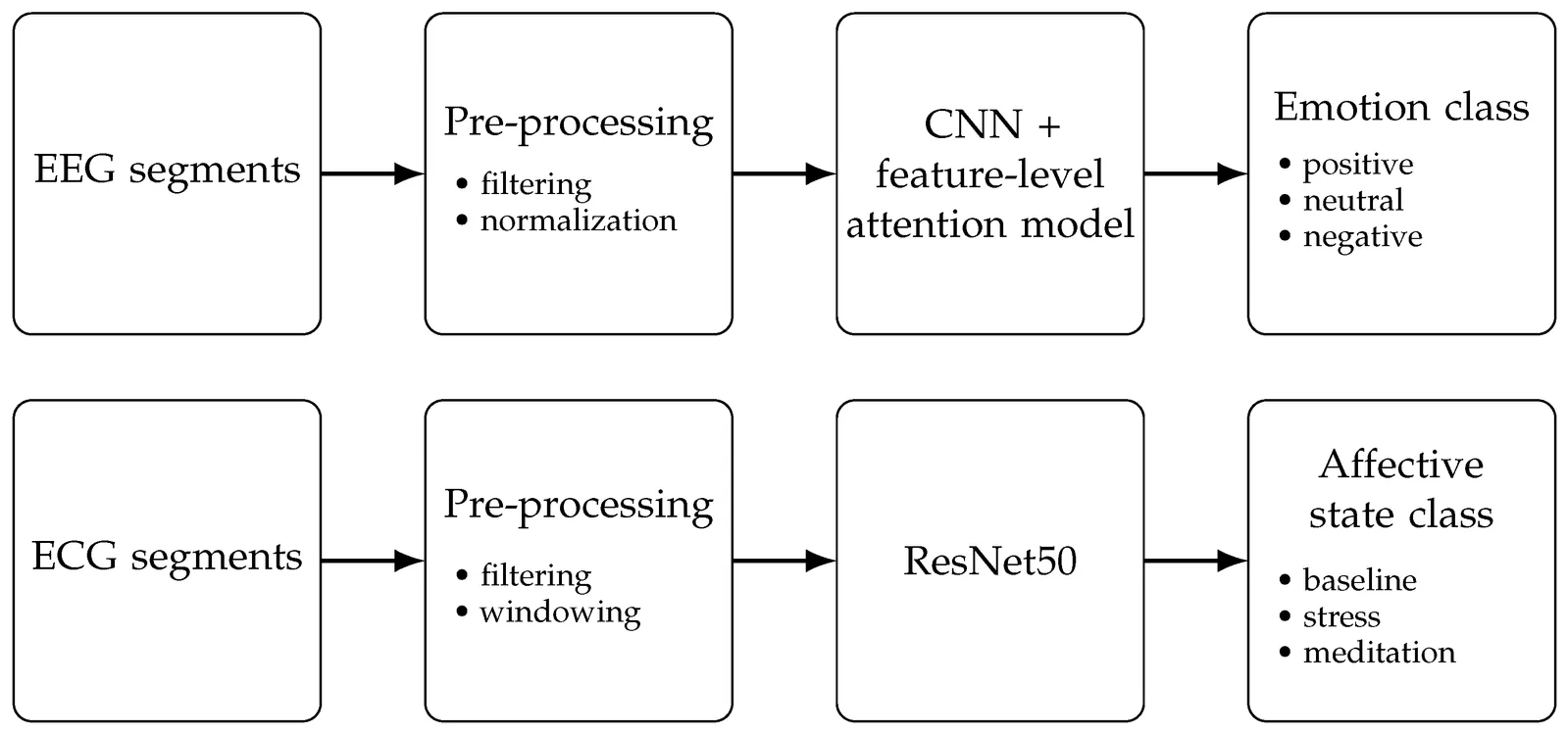
High-level deep learning classification pipelines for EEG- and ECG-based affective state recognition.

Deep learning alleviates the limitations associated with feature engineering approaches through learning task-relevant representations directly from raw or lightly processed physiological signals. In the proposed framework, DL models are trained on fixed-size segments and can handle modality-specific pipelines for affective state recognition using EEG and ECG signals, as shown in  Figure 3.

Convolutional neural networks (CNNs) have shown particular promise where physiological signals have natural structures, such as multichannel time windows or two-dimensional structures (e.g., time-frequency or image-like structures). CNNs can effectively use these structures to extract localized features, which can then be composed hierarchically to provide robust classification performance by learning time-series segments and then projecting them to structured inputs.

With regard to using transfer learning with ResNet50 to address ECG-based stress and affective states, optimization issues associated with DL architectures are effectively overcome using ResNet50, which uses skip connections, also known as residual connections. Mathematically, rather than learning a direct function H(x) using a deep neural network, each “residual block” learns a residual function:

(3) F(x)=H(x)−x⇒H(x)=F(x)+x.

As suggested in Equation (3), the identity shortcut helps stabilize the gradient flow in the backpropagation process, allowing deep convolutional neural networks to serve as effective feature extractors in the case of scarce labeled physiological data.

In the case of emotion recognition using an EEG, feature-level attention mechanisms are used to selectively emphasize the relevant components while suppressing the irrelevant components in the learned feature representation. This consideration is especially important in the case of an EEG, as the emotion-related components are typically weak, multichannel, and prone to artifacts.

### 3.4. Beyond 5G IoT Networks

Beyond 5G (B5G) solutions provide a more advanced integration of intelligence, edge computing, and adaptive network management into IoT networks. Conventional 5G IoT systems currently include reliability, reduced latency, and massive connectivity, yet B5G systems promise to dynamically optimize network behavior by adapting to varying traffic conditions, device densities, and application requirements, which allows efficient resource usage in B5G networks. The total available communication resources, such as bandwidth and time, can be dynamically shared among a large number of devices, and latency-sensitive data can be prioritized.

The achievable data rate of a wireless link is fundamentally bounded by the Shannon capacity and can be expressed as

(4) $$C=B\log_{2}\left(1+\gamma \right),$$

where *B* is the available bandwidth and γ is SNR. B5G systems are expected to operate across a wide range of spectrum bands, including sub-6 GHz, millimeter-wave (mmWave), and terahertz (THz) frequencies. This will provide significantly higher bandwidth compared to existing cellular systems, data rates approaching 1 Tbps, and end-to-end latency reduced to the order of sub-millisecond levels.

In B5G IoT systems, end-to-end latency can be described as the sum of the transmission delay, queuing delay, processing delay at edge/cloud nodes, and propagation delay. Simplified versions of end-to-end latencies of edge- and cloud-based systems can be expressed as in the Narayanan et al. [73] study:

(5) $$L_{\mathrm{edge}}=\frac{n_{b}}{R}+\frac{n_{b}n_{cpu}}{f_{\mathrm{edge}}},$$

(6) $$L_{\mathrm{cloud}}=\frac{n_{b}}{R}+\tau_{\mathrm{cloud}},$$

where $n_{b}$ represents the number of bits, *R* is the data rate based on Equation (4), $n_{cpu}$ is the number of CPU cycles per bit, $f_{\mathrm{edge}}$ is the clock frequency of the edge processor, and $\tau_{\mathrm{cloud}}$ represents the additional delay caused from the distance between gateaway and the cloud server. Equations (5) and (6) explicitly show that edge latency is jointly determined by transmission and processing delays. As can be seen from the equations, unlike edge-based systems, cloud latency is less sensitive to computational complexity, yet it is significantly affected by long-distance network delays. This implication emphasizes the importance of edge computing to reduce end-to-end latency in B5G IoT systems.

Considering these advancements, 6G communication systems, offering higher data speeds, higher reliability, higher connectivity, reduced latency, and fully integrated AI-based networks, aim to enhance the current infrastructure. The comparison of 5G/6G IoT parameters are given in  Table 1 [5,74].

6G networks have the potential to enhance the capabilities of 5G by operating in higher frequency bands along with more complex and sensitive propagation environments. In this context, accurate channel modeling is critical for understanding the performance limitations of 6G IoT systems under realistic environmental conditions.

## 4. Biosensor-Based Emotion Recognition with Feedback Control for Real-Time Healthcare over 6G IoT Networks

**Figure 4:**
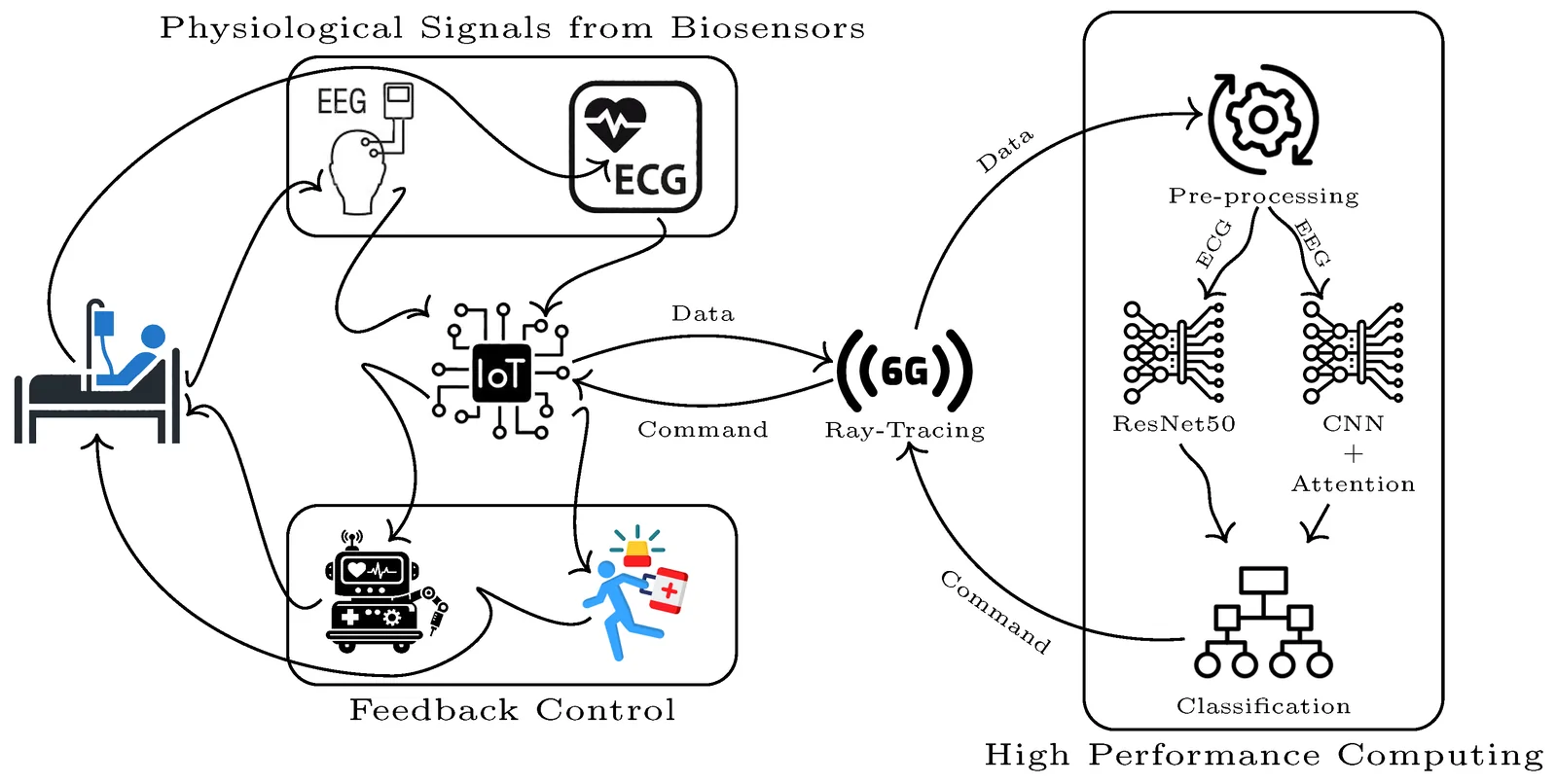
System architecture of the proposed framework.

In this study, we propose a framework for biosensor-based emotion recognition with feedback control in real-time healthcare over 6G IoT networks, as illustrated in  Figure 4. Physiological signals are collected from patients in intensive care units and transmitted securely and rapidly via IoT devices over the 6G band to a central system, where they are quickly processed using DL techniques on high-performance computers. This allows healthcare monitoring systems to facilitate emergency interventions, either by healthcare professionals or through autonomous mechanisms. Although the figure illustrates the proposed framework, the workflow presented in this study is trained and validated using a pre-existing dataset. The following subsection details the system architecture and workflow.

### 4.1. Overview

In this research, EEG and ECG signals are used for affective state recognition using modality-specific DL architectures. The EEG signal is used for three-class emotion recognition, with an attention mechanism incorporated into the architecture for highlighting the EEG patterns. The ECG signal is used for the recognition of stress and affective state, with the problem formulated as a four-class classification problem using transfer learning with a ResNet50 architecture after the transformation of the ECG signal into an image-like representation using the chest sensor.

This section presents the datasets used, pre-processing techniques, classification models, and a detailed methodology related to 6G communication.

### 4.2. Datasets

The SEED (EEG) [75,76] and WESAD (ECG) [58] datasets were treated as independent data sources in this study, as they were collected from different participant populations under distinct experimental protocols. Since these datasets do not provide synchronized EEG and ECG recordings from the same individuals, each dataset was analyzed and evaluated separately within the context of its respective experimental framework. SEED and WESAD datasets are publicly available in SJTU [77] and the UCI Machine Learning Repository [78], respectively.

EEG experiments utilize the SJTU Emotion EEG Dataset (SEED), a publicly available dataset developed by the Brain and Cognitive Machine Intelligence (BCMI) Laboratory at Shanghai Jiao Tong University [75,76]. The SEED dataset, which was utilized for this study, consists of EEG recordings obtained from a sample of fifteen participants (seven males and eight females, with an average age of 23.27 years ± 2.37 years) [75,76]. The participants attended three distinct sessions. The approximate time interval between each session was one week. During each session, participants were shown 15 Chinese film clips, each approximately four minutes in duration. The clips were designed to stimulate one of three emotional responses (i.e., positive, neutral or negative). EEG signals were recorded by utilizing a 62-channel ESI NeuroScan system, operating at a sampling rate of 1000 Hz. These signals were then downsampled to 200 Hz for the purpose of analysis. The signals were band-pass filtered between 0.5 and 75 Hz.

The ECG experiments were carried out using the WESAD dataset, a multimodal benchmark for stress and emotion recognition. The WESAD dataset was recorded from 15 participants (12 males and three females), denoted by S2–S17, with an average age of 27.47 years ± 2.45 years, using both chest-worn and wrist-worn sensors. The experiments in this paper only utilized the data obtained from the chest-worn sensors, which provide synchronized physiological data sampled at 700 Hz.

The WESAD dataset [58] was obtained from recordings made in controlled experiments where participants underwent various affective states, including baseline, stress, amusement, and meditation. The recordings, therefore, provide a diverse corpus of physiological data that can be employed in developing stress recognition models. The WESAD dataset has 24.13 h of recordings, with an average recording time of 96.5 min per participant [58]. The recordings have eight synchronized physiological channels, including accelerometer data along the x, y, and z axes (ACC x, y, z), an electrocardiogram (ECG), an electromyogram (EMG), electrodermal activity (EDA), skin temperature (Temp), and respiratory data (Resp). The mean distribution of class labels in the WESAD dataset for all participants is shown in  Table 2.

Both datasets mainly consisted of young individuals based on the mean age values. Therefore, an age-based evaluation was not performed in our study. In addition, due to the imbalanced distribution of male and female participants, no gender-based analysis was conducted.

### 4.3. Pre-Processing

#### 4.3.1. Pre-Processing Pipeline for SEED Dataset

The dataset consisted of multichannel EEG signals recorded from 62 electrodes with a sampling frequency of 200 Hz. The EEG recordings obtained under three emotional conditions (positive, neutral, and negative) were first arranged into matrix form, where each row represents a temporal sample, and each column corresponds to an EEG channel.

Before segmentation, the pre-processed EEG recordings provided by the SEED dataset were utilized [75,76]. According to the dataset protocol, the signals were band-pass filtered, and power-line interference was removed during the original pre-processing stage. No additional artifact-removal procedure, such as ICA-based ocular artifact correction or threshold-based segment rejection, was performed in our study. After window extraction, each segment was normalized to reduce inter-subject and inter-session variability and to support stable model training. The resulting segment tensors were then used as input to the EEG classification model.

To transform the continuous EEG signals into samples appropriate for the classification model, a segmentation procedure based on a sliding window technique was applied with no overlapping. Sliding window segmentation is widely used in EEG analysis to capture short-term temporal dynamics of neural activity while preserving temporal locality [79]. In this study, a window length of 2 s was selected. Given that the sampling frequency is 200 Hz, each segment contains 400 (i.e., 200 Hz × 2 s) temporal samples. Since the EEG recordings include 62 channels, each extracted segment has a dimensionality of 400 × 62. The segmentation process was implemented using a sliding window with a shift length of 4 s (800 samples). Let x(t,c) denote the EEG signal at time *t* for channel *c*. Each segment $S_{i}$ is defined as

(7) $$S_{i}=x\left(t_{i}:t_{i}+L-1,:\right),$$

where $t_{i}$ represents the starting index of the $i^{\mathrm{th}}$ window, and L denotes the window length. The starting indices are determined by

(8) $$t_{i}=1+\left(i-1\right)\times S,$$

where *S* represents the shift length (800 samples). The segmentation process continues as long as

(9) $$t_{i}+L-1\leq N,$$

where *N* is the total number of samples in the EEG recording.

Through this procedure, the continuous EEG signals were divided into multiple short-duration segments that represent localized patterns of brain activity. This segmentation allows the learning model to capture temporal variations in EEG signals that are related to different emotional states. Separate structure files were created for the positive, neutral, and negative emotion classes to maintain a clear data organization. The segmented datasets were then saved in MATLAB .mat format using the -v7.3 option, which supports efficient storage of large datasets. These EEG segments were used as input samples for the three-class emotion recognition model.

#### 4.3.2. ResNet50 Pre-Processing Pipeline for WESAD Dataset

This subsection describes the pre-processing steps used to process the WESAD dataset’s chest sensor recordings to identify stress and affective states using ResNet50. Specifically, the data collected using a 700 Hz sampling rate from 15 subjects (S2 to S17) is used. Each recording contains eight synchronized channels (ACC x/y/z, ECG, EMG, EDA, temperature, and respiration), forming a multivariate time series of size (N,8).

Firstly, pickle files containing data from all subjects were loaded, and the eight signals were extracted. The signals were then divided into windows using a sliding window approach with a window size of 700 (1 s) and a stride of 350 (50% overlap). Each window is then assigned a class label using majority voting, where only three classes were considered: baseline, stress, and meditation. The amusement class is excluded from the classification task. The class labels were then mapped to consecutive integers to suit PyTorch (v2.5.1) training function.

Windows were then divided into training, validation, and testing sets using a fixed-proportion subject-wise split (subject-independent evaluation). The 15 participants were allocated into 10 training, two validation, and three test subjects by applying train_test_split twice with random_state=42: first, 70% of the subjects were selected for training, and the remaining 30% were reserved as a temporary pool; then, the temporary pool was split equally into validation and test sets (15% each). The resulting allocation was training subjects S3, S4, S5, S6, S8, S9, S10, S13, S15, and S17, validation subjects S11 and S16, and test subjects S2, S7, and S14. No subject appears in more than one split; consequently, all windows from a given participant belong exclusively to either the training, validation, or test set. This procedure is distinct from leave-one-subject-out cross-validation; the reported validation and test accuracies are computed once on the corresponding held-out subject groups. Channel-wise z-normalization is then performed using training set statistics, as shown in Equation (10).

(10) $$x^{\prime }=\frac{x-\mu }{\sigma }$$

For facilitating transfer learning with ResNet50, each window normalized to size (700 × 8) is mapped to a compact two-dimensional representation by transposing it to (8 × 700) and then resampling it to 64 × 64 by interpolation to generate a sensor map and then expanding it to three channels to match the RGB input format required by ResNet50. Finally, each array is converted to a PyTorch tensor and loaded into a DataLoader for mini-batch training.

### 4.4. Classification Models

The classification stage is formulated as a supervised multi-class learning problem for both modalities. In EEG-based emotion recognition, the model receives multichannel signal segments and learns representations that capture spatio-temporal dependencies across channels and time. An attention mechanism is incorporated to adaptively emphasize informative patterns, which helps improve robustness to noise and inter-subject variability.

In ECG-based stress or affective state recognition, the model processes windowed chest sensor signals that are converted into image-like tensors. These tensors are then analyzed using a ResNet50 backbone within a transfer learning framework. The final class probabilities are produced by a task-specific classification head, and the model is trained using a cross-entropy loss function.

#### 4.4.1. EEG-Deep Learning Adaptation: Attention Mechanism

In this study, a feature-level attention mechanism was integrated into the CNN architecture. This mechanism improves the discriminative capability of the learned representations. After the convolutional layers extract features from EEG segments, the representation is transformed into a feature vector using a fully connected (FC) layer (128 neurons). This vector is then passed through an attention module consisting of a FC layer and a sigmoid activation function, which generates attention weights for each feature dimension. These weights are applied to the original feature vector through element-wise multiplication. The weights produce a reweighted representation that emphasizes informative features and suppresses less relevant ones.

Mathematically, let $h\in R^{d}$ denote the feature vector obtained from the FC feature layer. The attention module then computes a set of attention coefficients $a\in R^{d}$, according to the following formulation:

(11) $$a=\sigma \left(W_{a}h+b_{a}\right).$$

Here, $W_{a}$ and $b_{a}$ denote the learnable parameters of the attention layer, and $\sigma \left(.\right)$ represents the sigmoid activation function. The attention-refined feature representation *z* is obtained by element-wise multiplication of a and h (*a* ∘ *h*), which scales each feature dimension according to its learned importance before the final classification layer.

**Figure 5:**
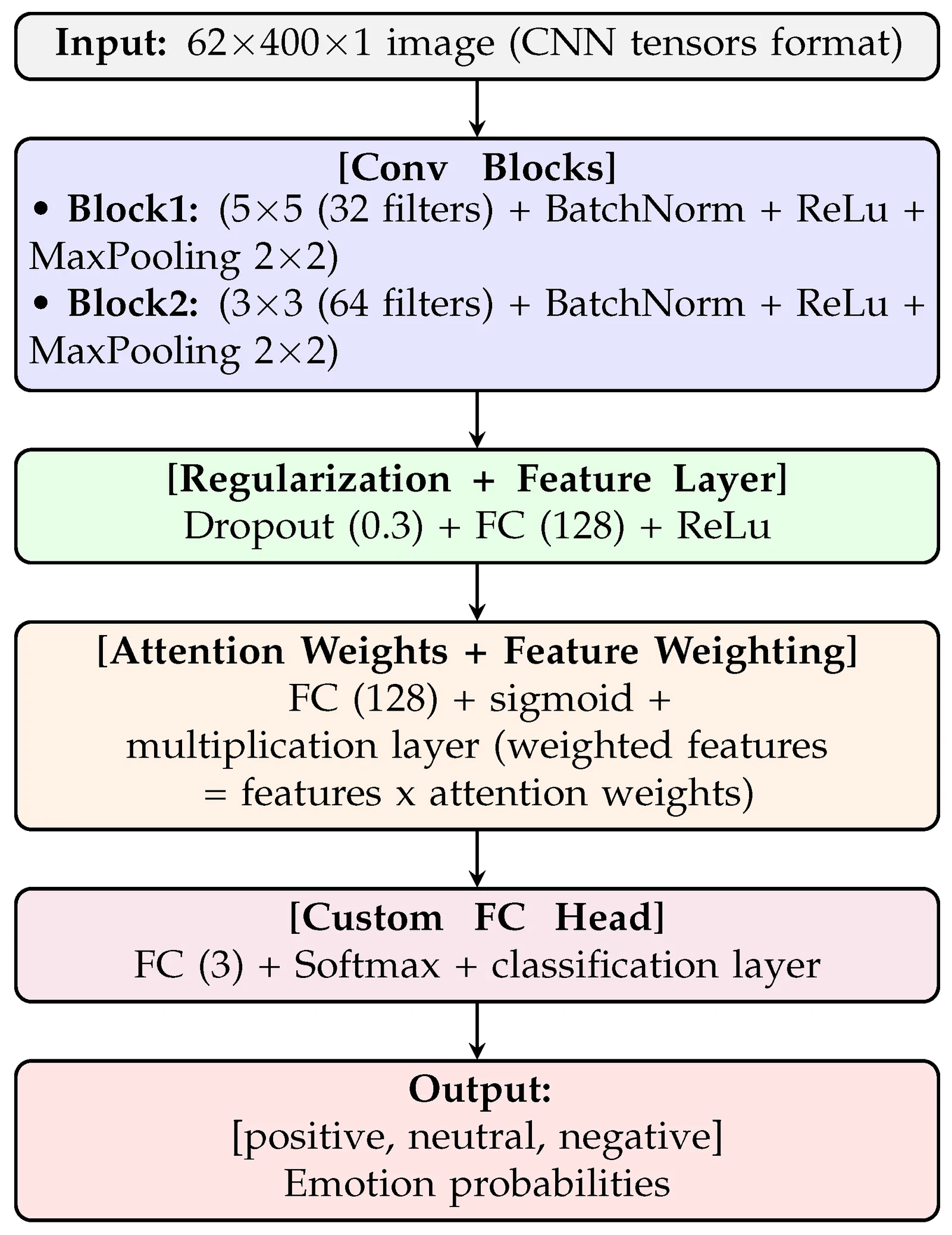
Flow diagram of the modified CNN + attention mechanism for SEED emotional state classification.

The attention module uses a sigmoid activation to assign independent weights to each feature, ranging values to (0, 1). This allows the model to emphasize informative features and give lower weights to less relevant features. Sigmoid activation processes features independently. Thus, this is useful for EEG-based emotion classification where multiple features can simultaneously have important information. In this way, the attention mechanism effectively reweights features to improve the learned representation. The integration of this attention-based feature reweighting mechanism allows the proposed model to focus on EEG patterns that are more relevant to emotional states before the final classification stage. By increasing the influence of informative features and reducing the contribution of less relevant components, the attention mechanism strengthens the discriminative capability of learned representations. This process helps to classify emotions more accurately. The flow diagram of the EEG-based emotional state classification framework is presented in Figure 5.

In this study, the dataset was initially randomly partitioned into an 80% training set and a 20% test set. Subsequently, 5-fold cross-validation was applied to the training set to ensure that the neutral emotion category was more adequately represented and could be learned more effectively. The resulting model was then saved and tested against the test data. The training parameters that were implemented in this study were as follows: the ADAM optimizer, 30 epochs, a mini-batch size of 64, a learning rate of 0.001, and shuffling at each epoch.

#### 4.4.2. ECG Branch: ResNet50 Transfer Learning

Deep learning is used to classify stress-related affective states from multimodal chest signals in the WESAD dataset. After transforming windowed time-series segments into compact 2D sensor map representations, CNNs are used to learn discriminative patterns across channels and time.

**Figure 6:**
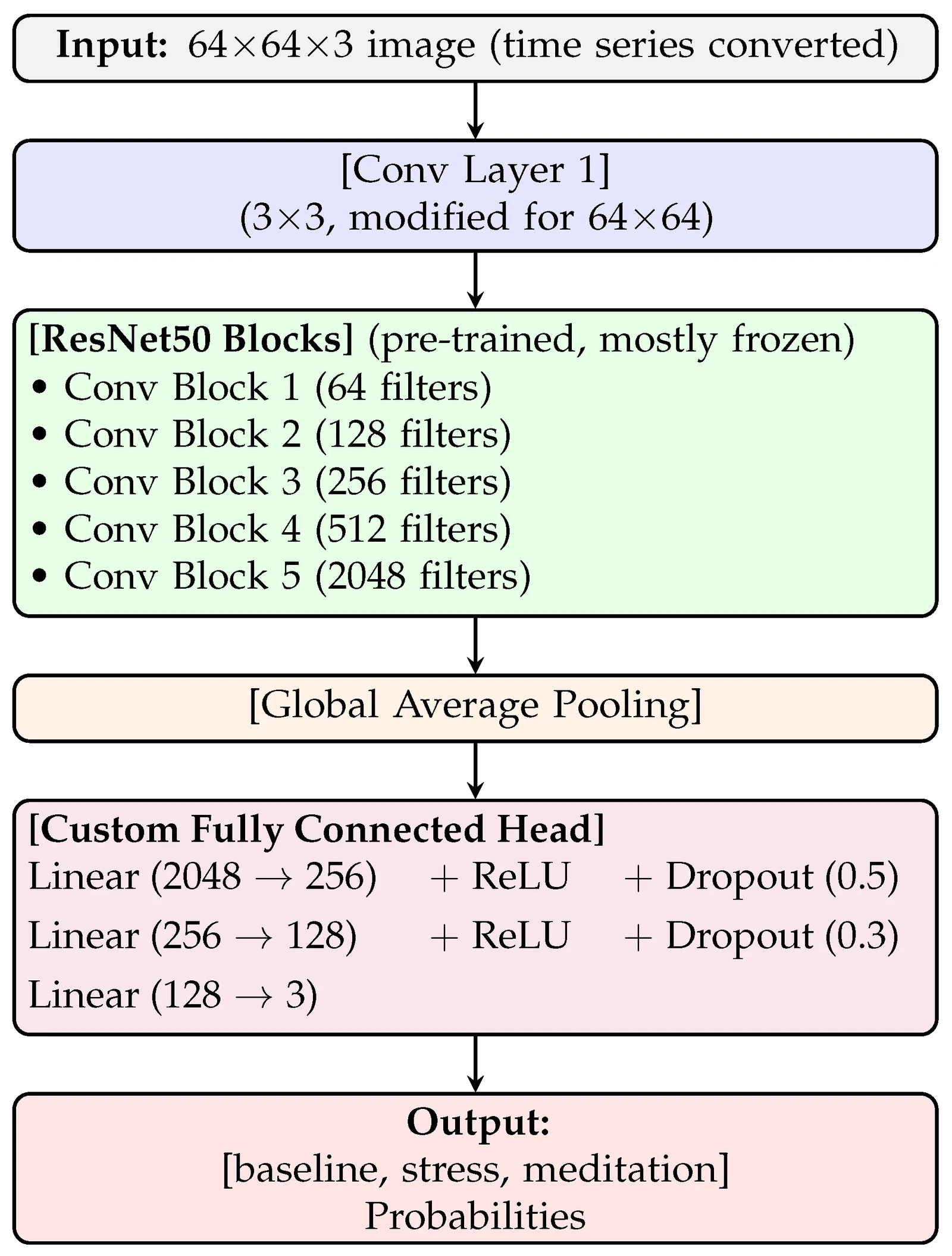
Flow diagram of the modified ResNet50 architecture for WESAD stress classification.

For ECG-based stress/affective state recognition, transfer learning with a ResNet50 backbone is used, as shown in Figure 6. The network is initialized with ImageNet-pretrained weights and fine-tuned to accept 64 × 64 RGB inputs from the physiological windows. Early layers may be frozen to preserve domain-agnostic feature extractors, while later layers are fine-tuned to learn domain-specific physiological features. A task-specific classification head is appended with a configuration of 2048 -> 256 -> 128 -> 4 with ReLU activations and dropout regularization.

Training is performed using an Adam optimizer with a cross-entropy loss function and standard regularization and learning rate scheduling techniques to ensure stable convergence and prevent overfitting.

### 4.5. Structure of 6G Ray Tracing-Based Large-Scale Fading for EEG and ECG Signals

The focus of this section is not on developing a new propagation model, but on examining the applicability of AI-assisted analysis of ECG and EEG data from biosensors within 6G-enabled healthcare communication architectures.

**Figure 7:**
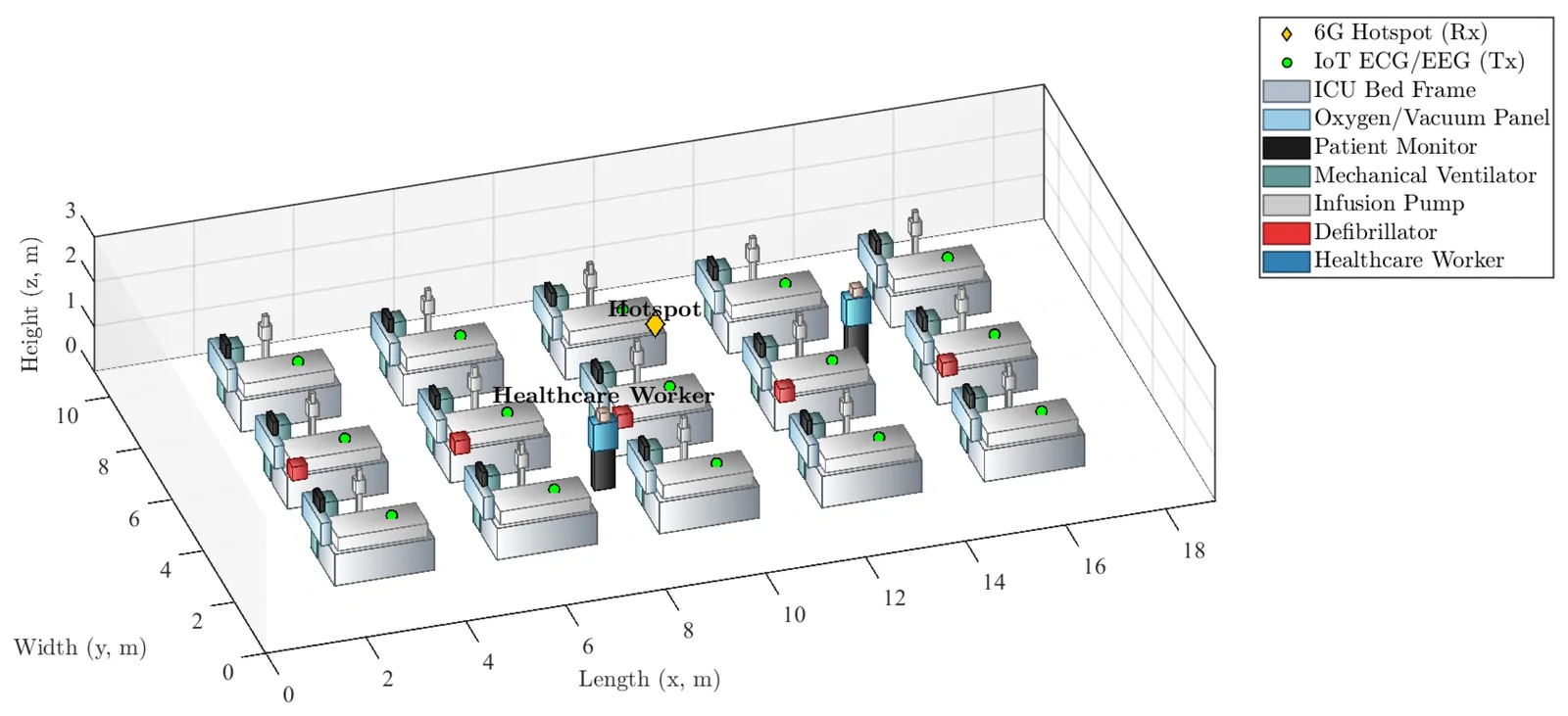
3D geometric model of a virtual hospital room.

In this study, a virtual hospital room, 19 m long, 11 m wide, and 3 m high, was created in a MATLAB R2024a simulation environment, containing 15 inpatients. The virtual room is composed of various materials such as glass, concrete, metal, and wood, and all of these are presented in Figure 7. Additionally, because ECG and EEG data from each patient’s IoT device in the room are transmitted wirelessly, there are 15 transmitters and one hotspot. This scenario, without many obstructions that could diffract or scatter the signal, allows for direct performance analysis of EEG and ECG signals transmitted over 6G mobile communication networks.

The channel modeling process consists of four main components: environmental modeling, a ray tracing algorithm, channel parameter analysis, and visual output presentation of the results. In the environmental modeling, the patient area, medical devices, furniture, and building elements within the hospital room were digitized in detail. The ray tracing algorithm models multipath propagation for 15 transmitters and a hotspot. Finally, the large-scale parameters of the channel are analyzed, and the resulting channel properties are presented as analytical outputs. Thus, the channel behavior in a 6G femtocell transmitting biomedical signals is modeled spatially and temporally.

The 3D layout of patient beds and the access point in the virtual hospital room is presented in Figure 7. The positions of the patient beds were randomly determined according to a normal distribution in the MATLAB environment. The location of the access point was chosen as the center of the room. IoT ECG and EEG sensors are indicated by green circles, and the access point is indicated by a yellow diamond. Objects in the room are shown in various colors. As presented in the legend, the mechanical ventilator is shown in cyan, the patient monitor is shown in dark gray, the infusion pump is shown in gray, the defibrillator is shown in red, and the healthcare worker is shown in navy blue. The virtual hospital room was designed to be identical to a real-world hospital room.

During the environmental modeling process, inpatients were placed at a height of 1 m off the floor, and the hotspot was placed at a height of 2.5 m off the floor. These values represent the standard patient bed height and hotspot height in a hospital room. The ray tracing algorithm used the 7.125–8.400 GHz frequency range in the FR3 band, which is intended for use in 6G mobile communication networks, and the 92–94 GHz frequency range in the millimeter-wave band. Because millimeter-wave signals have very short wavelengths and are subject to significant attenuation, directional antennas were preferred.

The output power of the IoT device transmitting ECG and EEG signals on the transmitter side is set to 1 mW. On the receiver side, the receiver sensitivity of the access point is set to −80 dBm. The 1 mW output power remains within the Specific Absorption Rate (SAR) limits while also providing low power consumption. The SAR refers to the energy absorption rate of an electromagnetic wave hitting the human body. The −80 dBm sensitivity represents the minimum power level at which the receiver can reliably detect a signal. These system parameters allow for a realistic modeling of the performance of 6G mobile communication networks in a virtual hospital room.

This study uses a ray tracing-based approach and a shooting and bouncing rays (SBR) method to model the path loss characteristics of 6G mobile communication networks during data transmission to a hotspot in a hospital room. The SBR method is based on the principles of geometric optics. Scattering analyses are performed in three stages. First, the SBR tracks the impact points and determines the electromagnetic fields sent from the transmitting antenna to these points. Then, Physical Optics (PO) defines the equivalent surface currents using surface equivalence theory. Next, the surface currents of all signals reflected after hitting any surface are summed and combined at the receiving antenna. Finally, the transmitting antenna radiates directly to the receiving antenna via the SBR, and these resulting fields are added to the sum obtained from the surface currents to create the final field solution at the receiving antenna. In the SBR method, the maximum number of reflections was selected as three. The receiver sensitivity was set at −80 dBm, and the thermal noise floor of the system was set at −100 dBm. In the SBR algorithm, reflections of order four or higher fall below the thermal noise floor. Therefore, rays that undergo four or more reflections are not considered, as tracking them would be meaningless. The virtual hospital room was modeled in 3D, and the dielectric properties of the walls, beds, medical monitors, and other furniture were modeled to be identical to the real world. All SBR-based ray tracing analyses were performed in the MATLAB R2024a environment.

Large-scale attenuation is modeled deterministically, while small-scale attenuation is modeled stochastically. Stochastic modeling has less computational complexity and models the effects of the environment by averaging random distributions. Deterministic modeling has higher computational complexity because the three-dimensional geometry and electromagnetic properties of the environment are fully defined. In this study, deterministic and stochastic modeling were used in a hybrid manner. Path loss was modeled deterministically with a near-range reference model. The log-normal shadowing effect was modeled stochastically with N=500 samples. Deterministic modeling determines how the signal will physically propagate. Stochastic modeling determines how much real-world randomness will statistically distort the signal. The antenna configurations, transmission powers, operating frequencies, and other fundamental parameters of the system used in path loss analyses are presented in Table 3. In addition, the frequency-dependent dielectric properties of the structural materials in the simulation environment are summarized in Table 4, based on ITU and IEEE technical reports.

#### Clinical Environment Path Loss Analysis in 6G Mobile Networks

Path loss refers to the decrease in power density of electromagnetic waves as they propagate through space from the transmitter to the receiver. In indoor environments, losses increase significantly due to physical obstacles. Therefore, in 6G mobile communication networks, path loss characterization in the FR3 and mmWave bands is an essential requirement for reliable channel models. Line-of-sight (LoS) propagation occurs when the transmitter and receiver antennas are directly aligned. Non-line-of-sight (NLoS) propagation occurs when the transmitter and receiver antennas are not directly aligned. In LoS conditions, signal attenuation varies depending on the distance, while in NLoS conditions, the signal undergoes reflection, diffraction, and scattering as it reaches the receiver. A hospital room contains numerous metallic medical devices, reflective surfaces, and human movement. This makes signal propagation extremely sensitive to the distance between the transmitter and receiver and the geometry of the environment. Both the SBR algorithm and the Monte Carlo algorithm were used in this study. SBR produced a deterministic path loss model by considering environmental conditions and the large-scale fading data obtained, while Monte Carlo modeled the log-normal shadowing effect stochastically. In the proposed scenario, the distance-dependent attenuation of signals transmitted from IoT-based ECG/EEG sensors to the hotspot is characterized by the following equation, based on a reference distance of $d_{0}=1$ m, according to the ITU-R P.1238-11 technical report:

(12) $$PL^{CI}\left(f,d\right)\left[dB\right]=FSPL\left(f,d_{0}\right)+10n\log_{10}\left(\frac{d}{d_{0}}\right)+X_{\sigma },$$

**Figure 8:**
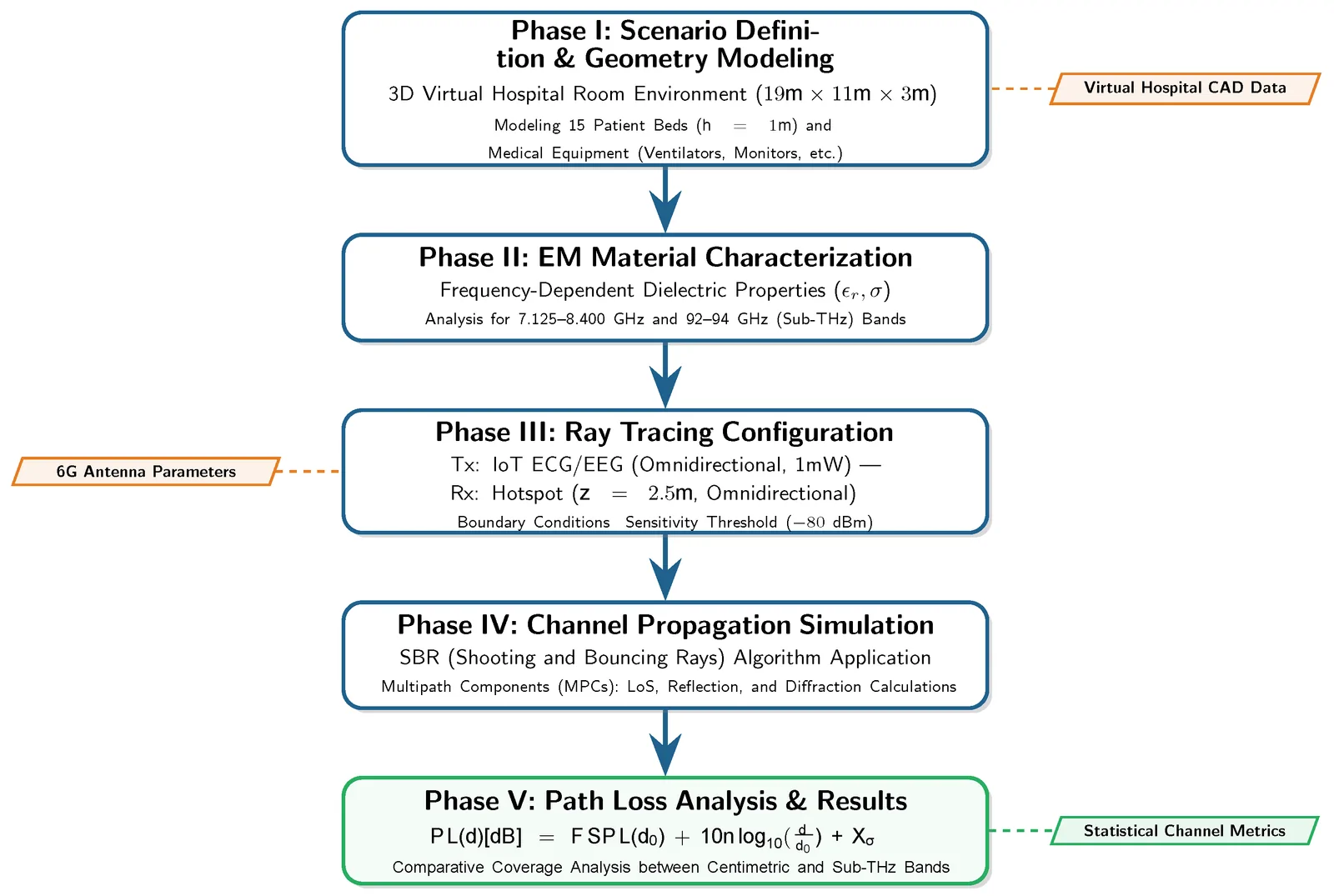
Methodological framework for the deterministic channel modeling of a 6G-based virtual hospital room.

Here, $PL^{CI}\left(f,d\right)$ represents the total path loss (dB) calculated at frequency *f* and distance *d*. *n* specifies the path loss exponent, which varies depending on the propagation characteristics of the medium. *d* represents the three-dimensional Euclidean distance between the transmitter and receiver. $X_{\sigma }$ represents the Gaussian random variable with zero mean and standard deviation σ, which statistically models the shadowing effects in the medium.

(13) $$FSPL\left(f,d_{0}\right)\left[dB\right]=20\log_{10}\left(d_{0}\right)+20\log_{10}\left(f\right)+20\log_{10}\left(\frac{4\pi }{c}\right),$$

where $FSPL(f,d_{0})$ represents the free-space path loss, *f* represents the frequency in Hz, and $d_{0}$ (usually 1 m) represents the reference distance. The path loss exponent varies between 2 and 4 in outdoor scenarios and between 1.6 and 6 in indoor scenarios, depending on the physical characteristics of the environment. The shadowing effect is the fluctuation in the received signal strength due to obstacles between the transmitter and receiver, and its standard deviation typically ranges from 3 dB to 12 dB. The flowchart for ray tracing-based path loss modeling in MATLAB for a virtual hospital room is presented in Figure 8. The close-in reference path loss model combines deterministic propagation principles with statistical variations. Therefore, it offers a higher prediction accuracy compared to classical models.

## 5. Experimental Evaluation

In this section, the proposed affective state recognition framework is evaluated from two different points of view. First, the classification results for the EEG-based emotion recognition branch on the SEED dataset and the ECG-based stress/affective state recognition branch on the WESAD dataset will be discussed using various metrics such as accuracy, loss curves, and confusion matrices. Second, a ray tracing-based characterization for a 6G IoT network in a virtual hospital scenario will be discussed.

### 5.1. EEG-Based Emotion Recognition Results

The performance of the proposed CNN+Attention mechanism-based EEG emotion recognition model on the SEED dataset is presented in this subsection. This is a three-class classification problem that includes positive, neutral, and negative states. The classification is achieved by using windowed multichannel EEG data that have been transformed into tensor forms to facilitate the proposed DL model.

**Figure 9:**
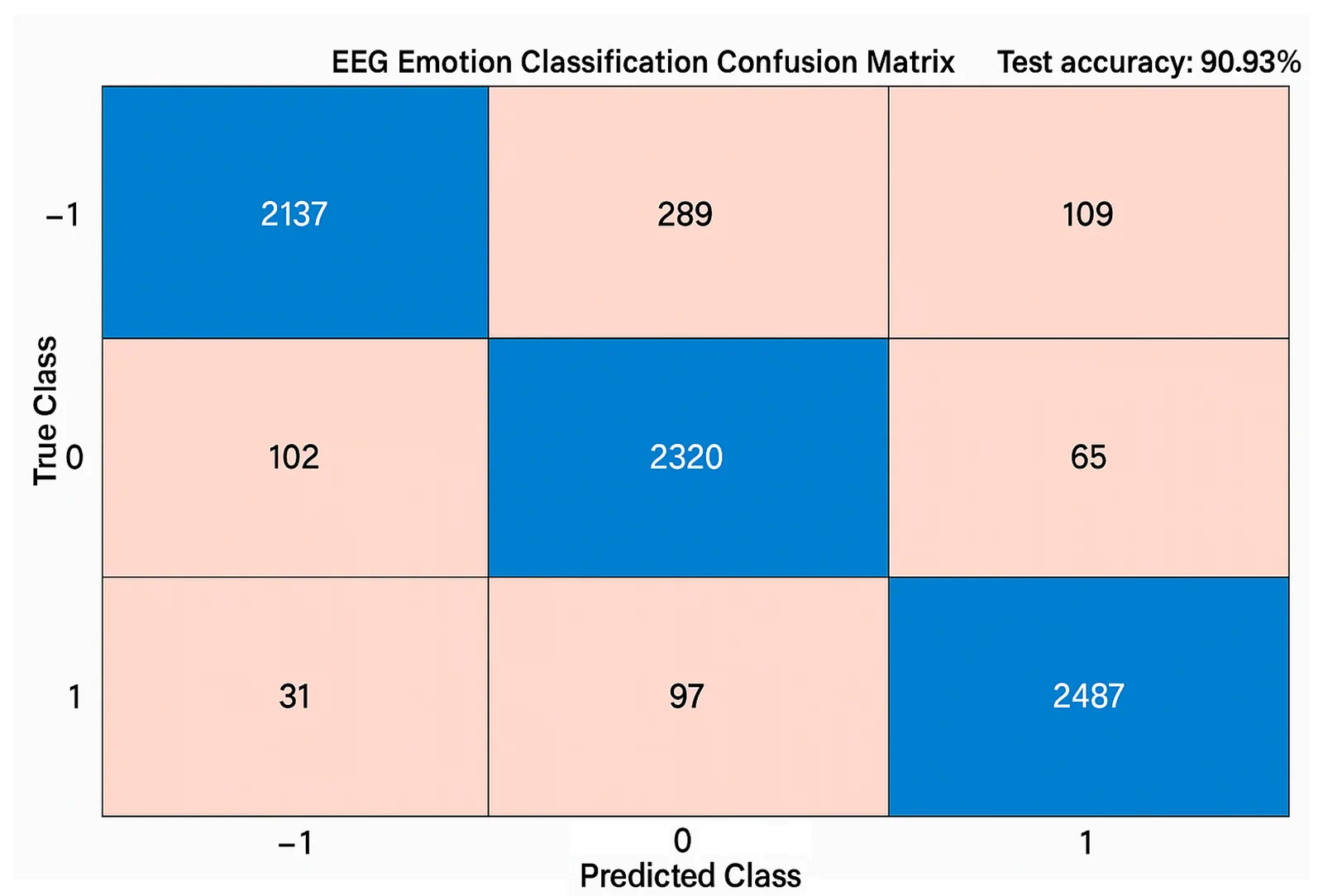
The results of emotional state classification using EEG signals. The label −1 represents negative emotions, 0 represents neutral emotions, and 1 represents positive emotions. The blue-colored diagonal cells represent the numbers of correctly classified samples, whereas the off-diagonal cells indicate the numbers of misclassified samples.

In the course of the training phase, a 5-fold cross-validation procedure was utilized, which resulted in a mean accuracy of 72.15% and a standard deviation of 1.31% in terms of accuracy. In the 5-fold cross-validation approach, the training data were divided into five subsets. These subsets are then updated using attention mechanisms, a process that ensures efficient weight updates within each subset. This improved the generalization capacity and accuracy rates of the proposed model when classifying test data. The results of the emotional state classification are shown in Figure 9.

Figure 9 shows that negative and positive emotions are sometimes misclassified as neutral, which may arise from the neutral class containing elements of both. Nevertheless, the model achieved a mean test accuracy of 90.93% across all three classes.

### 5.2. ECG-Based Stress and Affective State Recognition Results

This subsection describes the performance of the proposed ResNet50-based ECG stress/affective state recognition branch on the WESAD dataset. This is a three-class classification problem, including the baseline, stress, and meditation states, using windowed chest sensor data transformed into compact forms to facilitate transfer learning. The amusement class is excluded from evaluation to focus on the clinically relevant affective states. Table 5 shows subject-wise ECG classification performance on the test split.

The reported ECG accuracy figures should be interpreted in light of the subject-wise split procedure described in Section 4.3.2. The 82.26% value is the validation accuracy, and the 65.64% value is the test accuracy obtained on the fixed-proportion subject-wise split (10 training, two validation, and three test subjects), both computed on held-out subject groups. These values should not be interpreted as clinical ECG diagnosis accuracy (e.g., arrhythmia detection or cardiac disease diagnosis); rather, they reflect the difficulty of cross-subject generalization in affective state recognition.

In addition, the current experimental analysis is not stratified by demographic factors such as age and gender. Therefore, no age- or gender-dependent conclusions (e.g., which gender is “more responsible”) are drawn from these experiments. Future work will include subject-wise and subgroup-wise analyses to assess cross-subject generalization and demographic robustness.

It is important to note that the ResNet50 results reported in Table 5 were obtained from a single fixed-proportion subject-wise split, i.e., a single train/validation/test allocation. While this split is fully subject-independent and therefore evaluates cross-subject generalization to unseen participants, it does not constitute a full cross-validation study. To provide additional context on cross-subject generalizability, we also evaluated conventional machine learning baselines using a leave-one-subject-out (LOSO) cross-validation procedure on the same WESAD data. These LOSO baselines achieved a mean accuracy of 45.6% (Linear SVM) and 42.1% (Random Forest), which confirms that cross-subject affective state recognition is a challenging problem. The 65.64% test accuracy of the proposed ResNet50 on an independent subject-wise split therefore represents a meaningful improvement over these traditional ML cross-validation baselines. A full LOSO cross-validation of the deep ResNet50 model is reserved for future work, as training the network for every left-out subject is computationally expensive.

**Figure 10:**
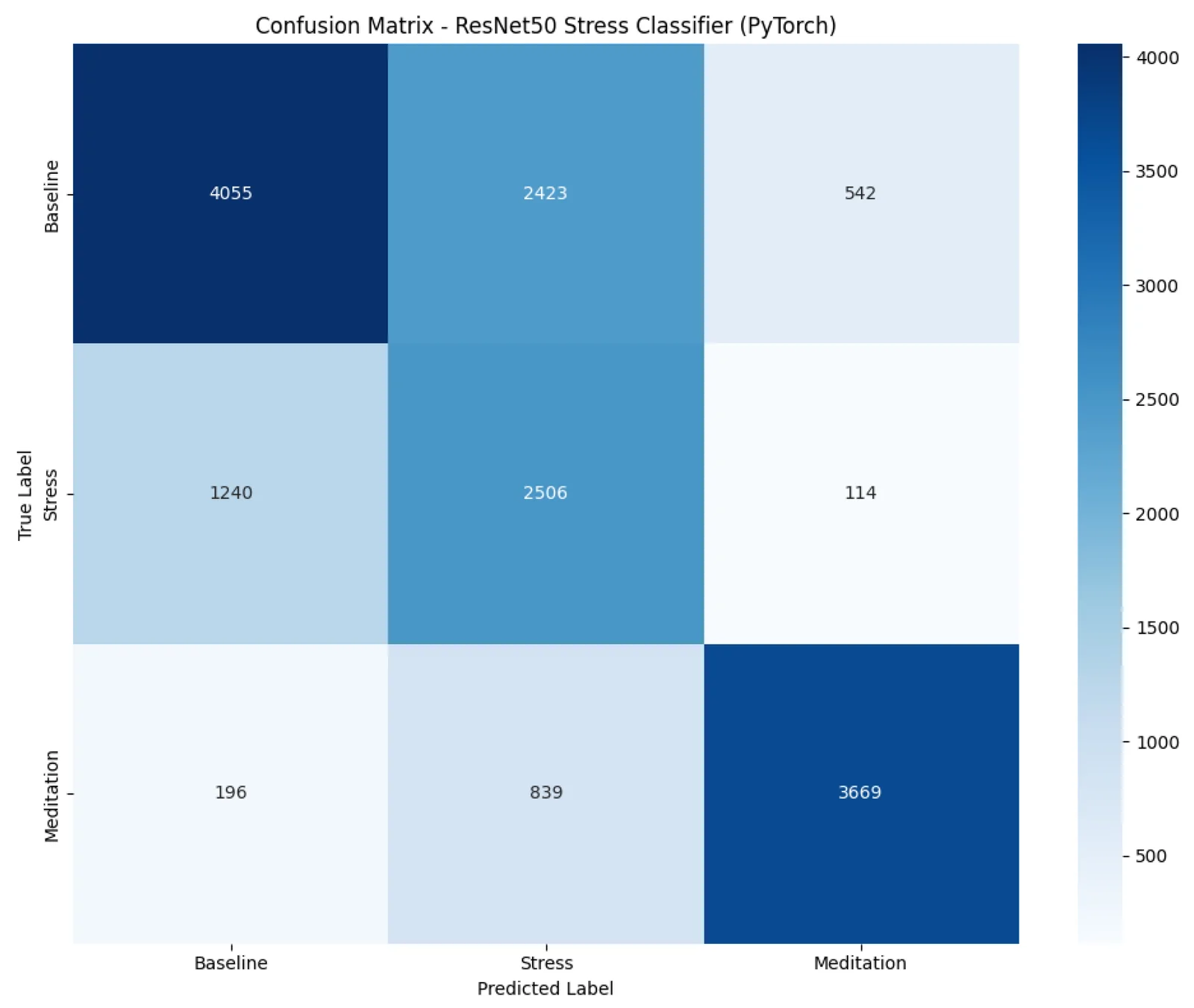
Confusion matrix for the ResNet50-based stress classifier on the WESAD dataset. The matrix illustrates the distribution of true versus predicted class labels across the three affective states.

Figure 10 corroborates the high separability of each class, as indicated by a strong diagonal structure in the confusion matrix with sparse off-diagonal elements corresponding to affective state confusions. This indicates that this ResNet50 model effectively learns stress-related physiological signals from windowed chest sensor data.

### 5.3. Path Loss Characterization of 6G IoT Networks

In this section, the propagation characteristics of the proposed 6G IoT healthcare framework based on biosensing data are analyzed using deterministic ray tracing simulations in a virtual hospital environment detailed in Section 4.5. We aim to evaluate the performance of the wireless channel for real-time transmission of EEG and ECG signals collected from wearable biosensors. Specifically, large-scale fading, spatial signal distribution, and temporal multipath behavior are investigated for both 7.75 GHz FR3 and 93 GHz mmWave frequency bands.

**Figure 11:**
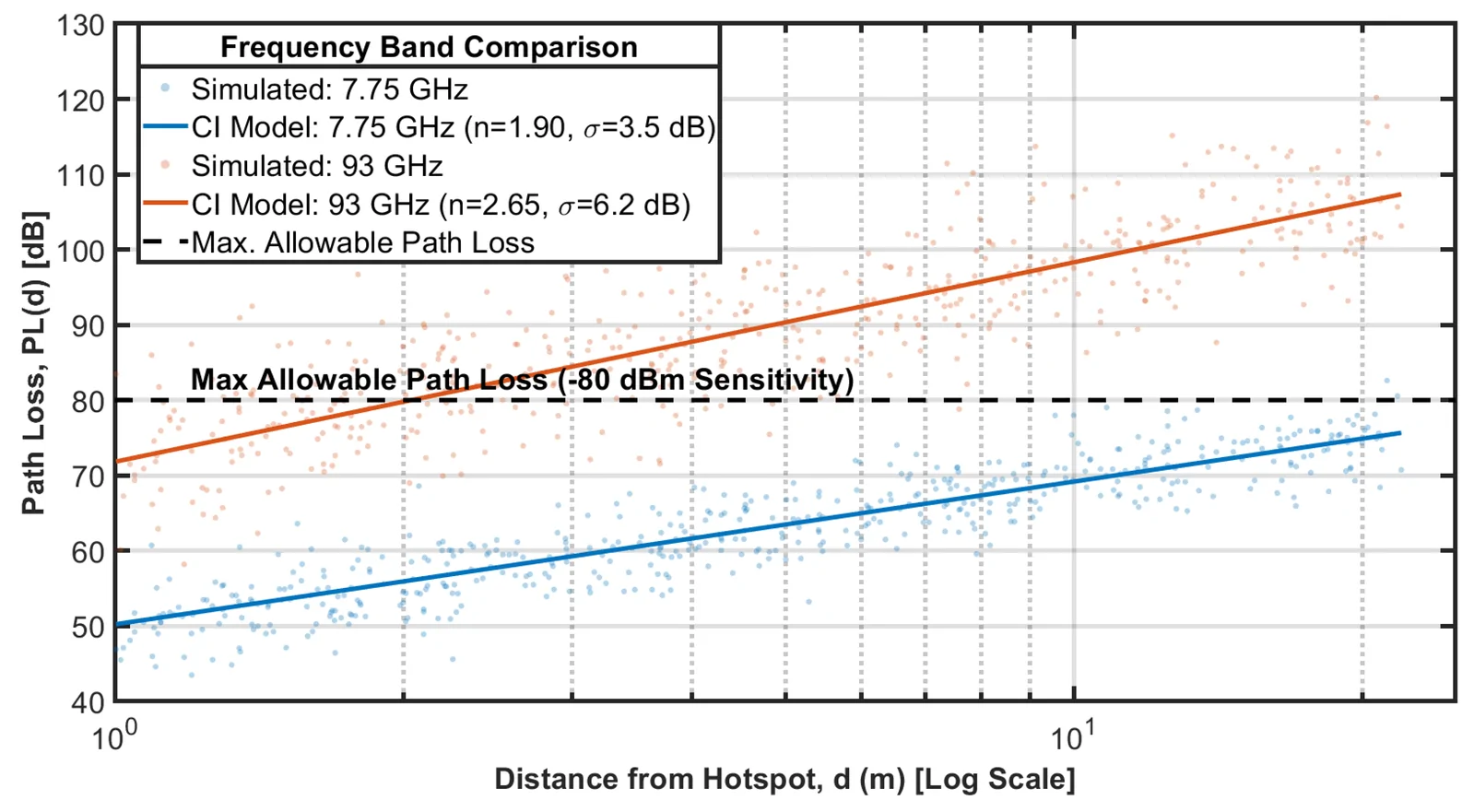
Close-in reference distance models for the 7 GHz band (7.75 GHz carrier frequency) and the 92 GHz band (93 GHz carrier frequency) for the virtual hospital room.

The distance-dependent path loss behavior is illustrated in Figure 11. Here, the simulated path loss results are compared with the close-in (CI) reference model, and the path loss variations according to the distance are observed for both frequency bands. As shown in the figure, the estimated path loss exponents are found as n=1.90 and n=2.65, with shadow fading standard deviations of σ=3.5 dB and σ=6.2 dB, for 7.75 GHz and 93 GHz, respectively. As can be seen from the results, signal attenuation increases significantly as frequency increases. At 7.75 GHz, the relatively low path loss exponent presents sufficient propagation conditions in indoor environments, due to stronger diffraction and penetration. On the other hand, as compatible with the known propagation properties of high-frequency bands, the higher path loss exponent at 93 GHz shows the high sensitivity of mmWave signals to blockage and material absorption [82].

Additionally, the distance dependency of path loss for FR3 and mmWave bands is compared according to the maximum allowable path loss threshold, determined by the receiver sensitivity of −80 dBm. As can be seen from Figure 11, path loss at 7.75 GHz remains well below the threshold across all distances until 22 m, the maximum range of the transmitter. At 93 GHz, after approximately 2 m, the allowed path loss level is exceeded. Without additional infrastructure, like beamforming and dense access point deployments, using only mmWave communication may not guarantee reliable coverage in indoor healthcare environments [83]. Despite the more robust main connectivity of FR3 bands, mmWave bands are more suitable for high-capacity and short-range links. The path loss data obtained in this study were compared and validated with existing studies in the literature in Table 6. It is clearly seen that the results obtained are in excellent agreement with similar studies.

**Figure 12:**
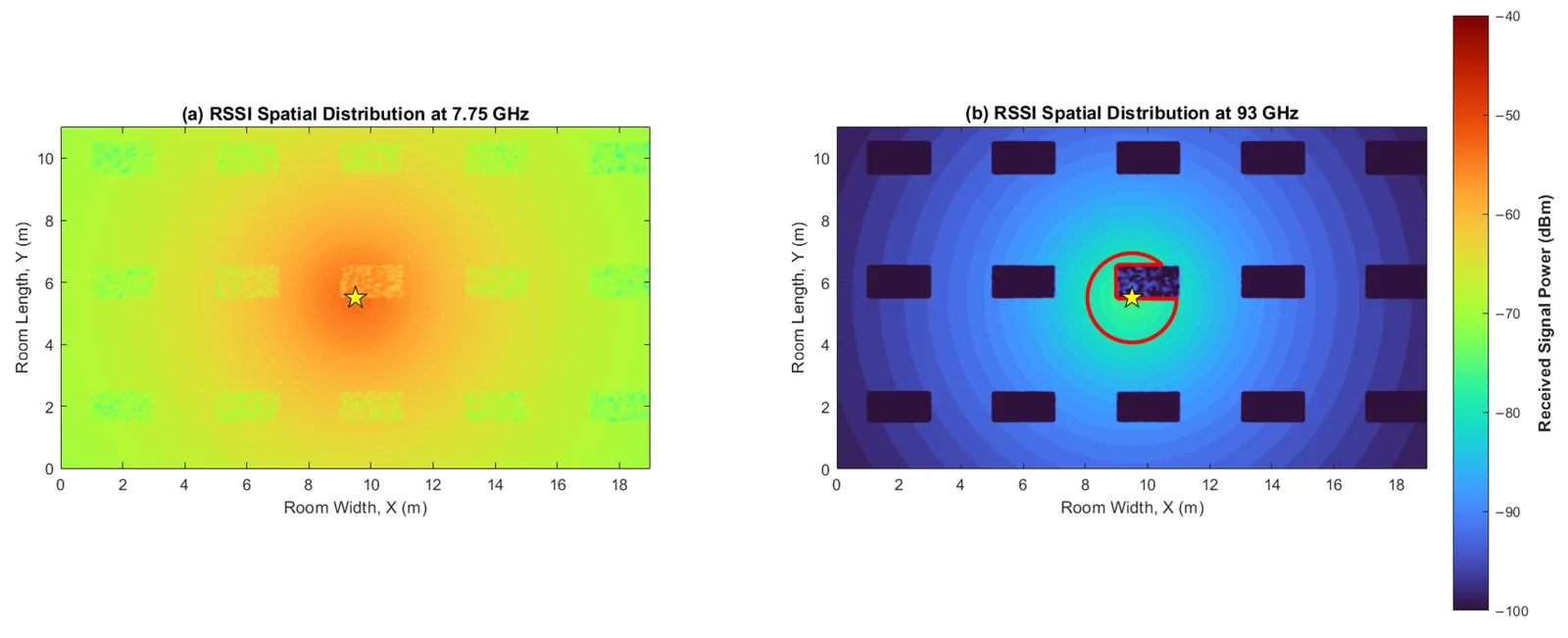
2D spatial distribution of received signal strength indicator (RSSI) for the virtual hospital room: (**a**) 7 GHz band (7.75 GHz carrier frequency); (**b**) 92 GHz band (93 GHz carrier frequency). The yellow star indicates the hotspot location, while the red circle denotes the boundary corresponding to the receiver sensitivity threshold of −80 dBm.

The 2D spatial distribution of the received signal strength indicator (RSSI) in the modeled virtual hospital room is given in Figure 12 for both FR3 and mmWave frequency bands. At 7.75 GHz frequency, the RSSI distribution is relatively smooth and regular in the room. The signals attenuate slowly as the distance from the hotspot increases. Obstacle effects, such as medical equipment and hospital beds, are limited. Thus, high spatial coverage and robustness against complex environmental conditions are obtained. The RSSI level stays above the sensitivity threshold, which allows reliable communication for IoT devices. On the other hand, the 93 GHz shows quite an irregular spatial pattern. Deep attenuation and shadowing effects are observed, especially on obstacles, as shown in Figure 12b. Signal coverage stays local around the hotspot, and small location changes result in large changes in RSSI. In biosensing-based real-time remote healthcare cases, continuous data transmission is very crucial. The lability of RSSI might cause the loss of packets and instant delays. Hence, precise network planning, directional antenna usage, beam management, and multiple connections may be required for mmWave bands.

**Figure 13:**
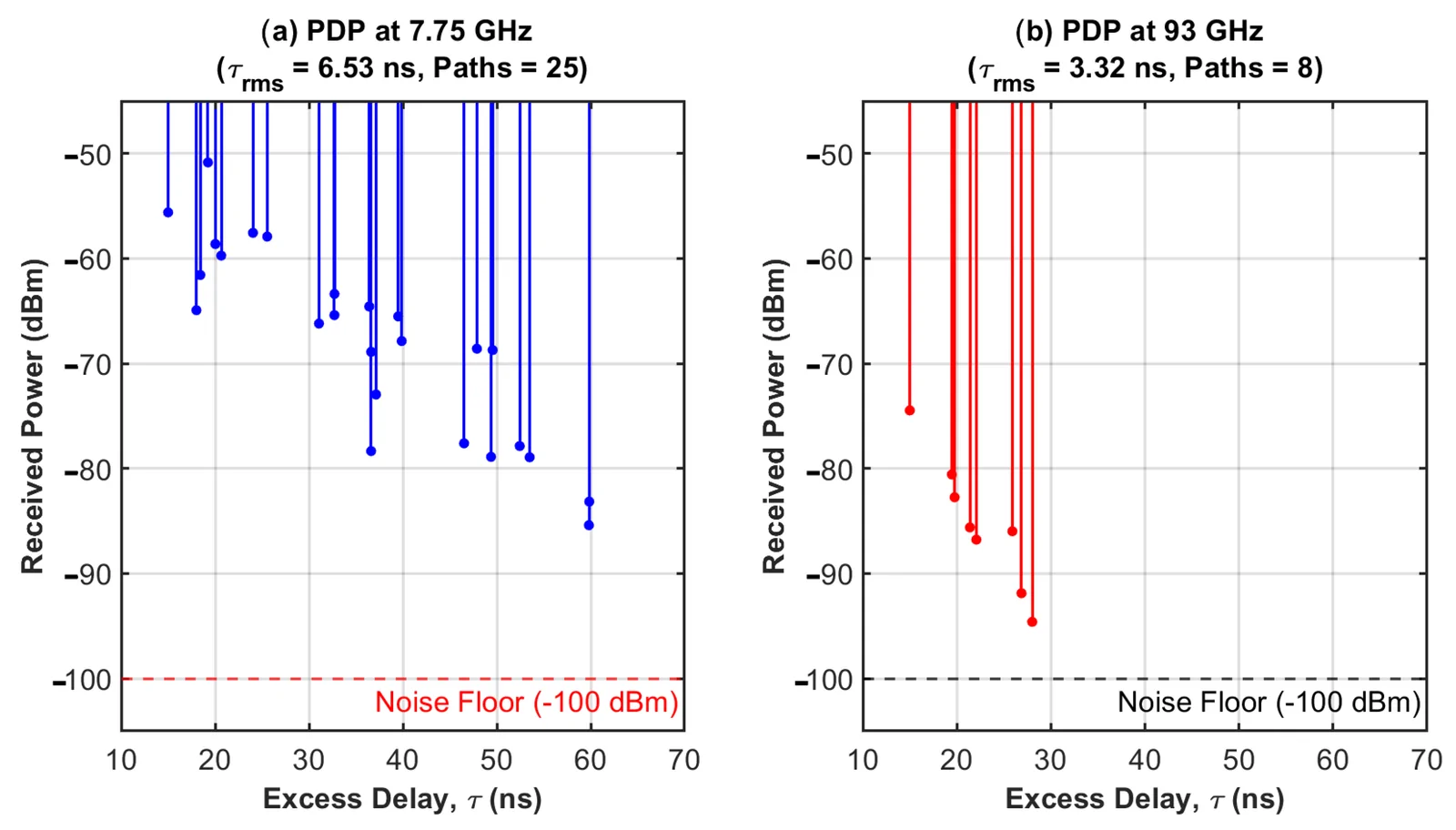
Power delay profiles (PDPs) showing temporal propagation characteristics for the virtual hospital room: (**a**) 7 GHz band (7.75 GHz carrier frequency); (**b**) 92 GHz band (93 GHz carrier frequency).

Figure 13 depicts the temporal propagation properties of the channel using power delay profiles (PDPs). At 7.75 GHz, a multipath structure with 25 significant propagation paths and a RMS delay spread of $\tau_{rms}=6.53$ ns is observed. The signals reach the receiver via multiple reflections and diffractions. Nevertheless, it increases the temporal dispersion and might introduce inter-symbol interference (ISI). Yet, it can provide robustness owing to multipath diversity. At 93 GHz, the PDP presents only eight dominant paths with a significantly lower delay spread of $\tau_{rms}=3.32$ ns. Here, only a limited number of strong paths contribute to the received signal. The reduced delay spread is advantageous for high data rate transmission, as it minimizes ISI. However, because of the lack of multipath diversity, the link may become more vulnerable to blockage and link failure. The PDPs obtained in this study were validated by comparing them with existing studies in the literature in Table 7. It is clearly seen that the results obtained are in excellent agreement with similar studies.

In addition, the correlation bandwidth is analyzed, which provides a measure of the frequency range over which the channel response remains highly correlated and is inversely related to the multipath delay spread. This inverse relationship results in a more frequency-flat channel behavior. Based on the ray tracing simulation results obtained in the virtual hospital environment, the channel gives a correlation bandwidth of 30.63 MHz for the 7 GHz band and 60.24 MHz for the 92 GHz band at a correlation level of 0.5, showing a moderate frequency selectivity for both bands. The 0.5 correlation level $B_{c,50\%}$ is approximated as follows:

(14) $$B_{c,50\%}\approx \frac{1}{5\tau_{\mathrm{rms}}}.$$

A trade-off between frequency bands can be emphasized from the results. In the FR3 band, a rich multipath, higher delay spread, and, consequently, robust, yet more complex, channel can be observed. mmWave presents sparse multipath and lower delay spread, resulting in high capacity, regardless of weak communication links. For the real-time EEG/ECG transmission case, which requires both reliability and low latency, these findings suggest that a hybrid communication strategy combining both bands can provide optimal performance.

Overall, the feasibility and limitations of 6G IoT-based wireless communication in indoor healthcare environments were evaluated using CI model fitting, which demonstrated that the proposed ray tracing framework accurately captures large-scale propagation behavior in a realistic hospital scenario. The obtained path loss parameters are found to be consistent with indoor propagation models reported in the literature, supporting the validity of the simulation approach. Second, the spatial and temporal analyses reveal that no single frequency band can simultaneously satisfy all requirements of real-time healthcare systems. While FR3 bands provide reliable coverage and stable connectivity, mmWave bands offer high data rates but require advanced deployment strategies. Considering the strict latency and reliability requirements of real-time remote healthcare applications, the results suggest that FR3 bands should be used for baseline connectivity and critical data transmission, such as continuous EEG/ECG streaming; mmWave bands should be leveraged for high-throughput, short-range communication such as burst data transmission or edge offloading. This hybrid approach aligns with the envisioned architecture of 6G IoT networks and ensures that the proposed biosensor-based emotion recognition system can operate effectively in realistic hospital environments.

## 6. Conclusions and Future Work

In this study, a framework for EEG and ECG wearable biosensor-based affective state analysis using deep learning over 6G IoT healthcare networks was proposed to support reliable and low-latency physiological signal transmission. Experimental results showed that the EEG (emotion recognition) and ECG (stress/affective state recognition) models achieved 72.15% (validation)/90.93% (test) and 82.26% (validation)/65.64% (test) accuracy, respectively. Additionally, the network provided RMS delay spread values of 6.53 ns for the 7 GHz band and 3.32 ns for the 92 GHz band, and correlation bandwidth values of 30.63 MHz for the 7 GHz band and 60.24 MHz for the 92 GHz band at a correlation level of 0.5. The simulation results demonstrate the feasibility of integrating wearable biosensor-based affective state analysis with 6G-oriented IoT healthcare communication frameworks.

One limitation of this study is that it was evaluated only on specific emotion and affective state classes under limited scenarios. Multiple emotion classes, heterogeneous user groups, and broader healthcare conditions have not yet been considered. Another limitation is that the 6G IoT network was evaluated only through ray tracing-based simulations and has not been validated on a real 6G infrastructure or under different network conditions. The deterministic ray tracing model includes the room structure, patient beds, human body properties, and medical equipment; it does not consider the time-varying blockage caused by moving people and equipment relocation. Nevertheless, the current simulation results demonstrate the potential applicability of the proposed framework for real-time affective state monitoring scenarios.

As future work, more comprehensive evaluations can be performed by including different age groups, health conditions, and heterogeneous physiological responses in the datasets. In addition, this study can be expanded to handle multiple affective state classes and more complex physiological state monitoring scenarios. Furthermore, incorporating additional wearable biosensors, such as GSR (Galvanic Skin Response), EMG (electromyography), and PPG (photoplethysmography), into the framework may further improve multimodal physiological analysis performance. Comprehensive measurement campaigns can be conducted to evaluate the model’s performance under real-world conditions. The combined latency and data loss arising from signal acquisition, pre-processing, data transmission, and the effect on classification accuracy can be evaluated through a hardware-based implementation and measurements in a real hospital environment. Also, combining ray tracing with time-varying and probabilistic wireless channel models could enable more realistic communication simulations. Finally, similar wearable biosensor and IoT-based approaches may be extended to other real-time affective state monitoring and assistive healthcare applications.

Moreover, future studies will consider stratified evaluation across demographic subgroups (e.g., age and gender) and subject-wise protocols (e.g., leave-one-subject-out) to avoid subject-specific leakage and to assess fairness and generalization under real-world deployment settings.

## Figures and Tables

**Table 1:** The comparison of 5G/6G IoT features.

Parameter	5G IoT	6G IoT
Data Rate	10–20 Gbps	100 Gbps–1 Tbps
Latency	1–5 ms	0.01–0.1 ms
Reliability	99.999%	99.99999%
Connection Density	$10^{6}$ devices/km^2^	$10^{7}$ devices/km^2^

**Table 2:** Mean label distribution in the WESAD dataset.

Label	Description	Proportion (%)
Not defined	Data outside labeled intervals	45.0
Baseline	Resting or neutral state	20.4
Stress	Trier social stress test phase	11.6
Meditation	Guided relaxation	13.7
Amusement	Positive emotional stimulus	6.5
Unknown (6)	Miscellaneous	0.9
Unknown (7)	Miscellaneous	0.9

**Table 3:** Simulation parameters.

Parameter	Value
Carrier Frequency	7 and 92 GHz
IoT Antenna Type	Omnidirectional
Hotspot Antenna Type	Omnidirectional
Transmitter Power	1 mW
Maximum Range of the Transmitter	22 m
IoT Height	1 m above ground level
Receiver Sensitivity	−80 dBm
Coverage Area	$19\times 11\;m^{2}$
Material	Concrete, glass, metal and wood
Scenario	Hospital room

**Table 4:** Dielectric properties and conductivity values of structural materials in the hospital room.

Material	Dielectric Constant ($\varepsilon_{r}$)	Conductivity (σ) [S/m]	References
Concrete	5.24	0.0462–0.7822	ITU-R P.2040-3 [80]
Glass	6.31	0.0036–1.3394	ITU-R P.2040-3
Wood (dry)	1.99	0.0047–1.0718	ITU-R P.2040-3
Metal	1.0	$10^{7}$	ITU-R P.2040-3
Human body (skin)	≈6.5–34.5	≈5.1–40	IEEE Std C95.1^™^-2019 [81]

**Table 5:** Subject-wise ECG classification performance on the WESAD test split.

Class	Precision	Recall	F1-Score	Support
Baseline	0.74	0.58	0.65	7020
Stress	0.43	0.65	0.52	3860
Meditation	0.85	0.78	0.81	4704
Accuracy	0.66			15,584
Macro avg	0.67	0.67	0.66	15,584
Weighted avg	0.70	0.66	0.67	15,584

**Table 6:** Comparison of the obtained path loss parameters with state-of-the-art indoor propagation models.

Reference	Frequency Band (GHz)	Scenario	Antenna Config.	Model	PLE (*n*)		Shadow Fading (σ, dB)
					LOS	NLOS	
[84]	6.75	Office/Hotspot	Omnidirectional	CI	1.40	2.42	LOS: 3.41/NLOS: 7.87
[85]	6.75	Factory (MakerSpace)	Omnidirectional	CI	1.39	1.78	LOS: 1.86/NLOS: 2.46
[86]	6.75	Office/Hotspot	Omnidirectional	CI	1.30	2.70	LOS: 3.50/NLOS: 9.20
[87]	7.0–20.0	Indoor Corridor	Omnidirectional	CI	Frequency-Dependent	Frequency-Dependent	Linear Variation
[88]	90.0	Office Corridor and Aircraft Hangar	Directional	CI	1.85	3.14	LOS: 2.98/NLOS: N/A
[89]	95.8	Conference Room	Omnidirectional	CI	1.38	N/A	LOS: 0.83/NLOS: N/A
This work	7.0	Hospital room	Omnidirectional	CI	1.90	N/A	LOS: 3.5/NLOS: N/A
	92.0				2.65	N/A	LOS: 6.2/NLOS: N/A

Note: N/A = not available.

**Table 7:** Comparison of simulated power delay profile characteristics with state-of-the-art measurement-based studies.

Reference	Frequency Band (GHz)	Scenario	Mean Excess Delay (μs)	RMS Delay Spread (ns)	Peak Power (dBm)
[90]	93	Indoor (Water Bottle Blockage)	0.066	N/A	−17.82
		Indoor (A4 Paper Blockage)	0.016	N/A	−17.03
		Indoor (Plastic Bag Blockage)	1.000	N/A	−20.69
		Indoor (Sponge Blockage)	1.567	N/A	−21.66
[84]	6.75	Office/Hotspot	N/A	19.3 (Dir. LoS)/33.7 (Omni LoS)	N/A
[85]	6.75	Factory (MakerSpace)	N/A	14.0 (Dir. LoS)/30.3 (Omni NLoS)	N/A
[91]	90	Airport Baggage Claim Area	N/A	20.5 (LoS)/24.0 (NLoS)	N/A
[92]	105	Meeting Room/Office (WPAN)	N/A	<5	N/A
[93]	4.4–6.5	In-Vehicle (Engine Compartment)	N/A	0.39–2.55	N/A
	99–101.5		N/A	0.55–1.30	N/A
This work	7.0	Hospital Room	0.02478	6.53	−53.52
	92.0		0.01679	3.32	−76.0

Note: Dir. = directional, Omni = omnidirectional, WPAN = Wireless Personal Area Network, and N/A = not available.

## Data Availability

The datasets used in this study are publicly available. The SEED EEG dataset and the WESAD ECG dataset were used for the experimental evaluations. The corresponding acquisition details and references are provided in Section 4.2.
